# hsa_circ_0003596, as a novel oncogene, regulates the malignant behavior of renal cell carcinoma by modulating glycolysis

**DOI:** 10.1186/s40001-023-01288-z

**Published:** 2023-09-02

**Authors:** QingZhi Xie, FuQiang Qin, LiHui Luo, ShaoQuan Deng, Ke Zeng, YunChou Wu, DunMing Liao, Lin Luo, KangNing Wang

**Affiliations:** 1 Department of Urology Surgery, The First Affiliated Hospital of Shaoyang University, Shaoyang, 422000 Hunan China; 2 Department of Personnel Section, The First Affiliated Hospital of Shaoyang University, Shaoyang, 422000 Hunan China; 3 Department of Urology Surgery, Xiangya Hospital Central South University, Changsha, 410008 Hunan China

**Keywords:** hsa_circ_0003596, Glycolysis, Renal cell carcinoma, miR-370-5p; PRKCSH

## Abstract

**Background:**

This research was planned to analyze hsa_circ_0003596 (circCOL5A1) and glycolysis-focused mechanisms in renal cell carcinoma (RCC).

**Methods:**

circCOL5A1, miR-370-5p, and PRKCSH levels were determined in RCC tissues and selected cell lines by RT-qPCR and/or Western blot. RCC cells after corresponding transfection were tested by colony formation assay, EdU assay, Transwell assay, and flow cytometry to analyze cell proliferation, invasion, migration, and apoptosis. Meanwhile, glycolysis in cells was evaluated by measuring glucose consumption, lactic acid, and ATP production, as well as immunoblotting for HK2 and PKM2. In addition, circCOL5A1 knockdown was performed in animal experiments to observe tumor growth and glycolysis. Finally, the ceRNA network between circCOL5A1, miR-370-5p, and PRKCSH was studied by luciferase reporter assay and RIP experiment.

**Results:**

circCOL5A1 and PRKCSH were highly expressed and miR-370-5p was poorly expressed in RCC. circCOL5A1 knockdown depressed RCC proliferation, invasion, migration, and glycolysis, and enhanced apoptosis. circCOL5A1 competitively adsorbed miR-370-5p. Artificial upregulation of miR-370-5p saved the pro-tumor effect of circCOL5A1 on RCC cells, as evidenced by suppression of tumor malignancy and glycolysis. miR-370-5p targeted PRKCSH. PRKCSH overexpression contributed to a reversal of the anti-tumor effect of circCOL5A1 silencing. Silencing circCOL5A1 inhibited RCC tumor growth and glycolysis.

**Conclusions:**

circCOL5A1 regulates the malignant behavior of RCC by modulating glycolysis.

**Supplementary Information:**

The online version contains supplementary material available at 10.1186/s40001-023-01288-z.

## Introduction

Renal cell carcinoma (RCC) originates from tubular epithelial cells of the kidney and is highly malignant and heterogeneous [1]. In most cases, this is a silent disease until it reaches an advanced stage and new effective biomarkers need to be found in all areas from detection to post-treatment surveillance [2]. RCC exhibits significant alterations in cell metabolism, so that tumor cells preferentially induce the hypoxic response pathway through glycolysis rather than normal oxidative phosphorylation for energy [3]. Defects occur in metabolic pathways from glycolysis to mitochondrial function and affect not only tumor cell function but also the local environment [4]. Given that, this research investigated the molecular mechanism underlying glycolysis in RCC.

circRNAs, a novel type of regulatory RNA, have been implicated in the progression of human cancers, including but not limited to RCC [5]. Importantly, it has been highlighted that circRNAs exert greatly to optimize diagnosis and improve patient survival in RCC [6–8]. As for circCOL5A1, only some papers have explored its action in keloid progression, but few in tumorigenesis. Hence, the research mainly focused on the molecular mechanism of circCOL5A1 in RCC.

miRNAs have the advantage of promoting early diagnosis of RCC [9], and some miRNAs directly targeting glycolytic enzymes are downregulated in cancer, strongly influencing the Warburg effect [10]. The correct identification of the potential interaction between circRNAs and miRNAs improves the insight into disease pathogenesis, as well as provides a niche for diagnosing and managing the disease [11]. miR-370-5p has been widely accepted as a tumor suppressor [12–14]. Definitely, the action of miR-370-5p in RCC progression, especially in the field of glycolysis, is uncertain.

The current study picked miR-370-5p as the downstream target of circCOL5A1 and mainly investigated how the interaction between circCOL5A1 and miR-370-5p regulates RCC progression in part through modifying glycolysis.

## Materials and methods

### Clinical samples

Seventy-three pairs of RCC tissues and matching paracancer tissues were collected from RCC patients who underwent radical nephrectomy or nephrectomy (2017–2020) in The First Affiliated Hospital of Shaoyang University. All cases were confirmed by two independent histopathologists. No radiotherapy, chemotherapy, or other adjuvant therapy was performed prior to surgery. The tissues were stored at − 80 °C for subsequent RNA or protein extraction. All patients provided informed consent and the study design was approved by the ethics Review Committee of The First Affiliated Hospital of Shaoyang University.

### RT-qPCR

After extracting RNA from cell lines and tissues based on Trizol (Invitrogen), cDNA was generated using PrimeScript RT kit (Takara) and tested with the SYBR Premix Ex Taq kit (Takara, Dalian, China) to analyze circRNA/mRNA expression or with TaqMan MicroRNA Assays (Invitrogen) to measure miRNA expression. U6 and GAPDH were endogenous control genes, respectively. Gene expression was calculated using 2^−ΔΔCt^. Primer sequence information is given in Table 1.

**Table 1:** Gene sequence primers

Genes	Sequences (5ʹ–3ʹ)
circCOL5A1	Forward: 5ʹ-CCTAACCAAGGATGCTCCAGG-3ʹ
	Reverse: 5ʹ-GGCCCCCTTCGGACTTCT-3ʹ
miR-370-5p	Forward: 5ʹ-GCAGGTCACGTCTCTGC-3ʹ
	Reverse: 5ʹ-TGGTGTCGTGGAGTCG-3ʹ
PRKCSH	Forward: 5ʹ-TGCCTTCAAGGAGCTGGATG-3ʹ
	Reverse: 5ʹ-AAAGAGGTGGCGTCTGTCTG-3ʹ
U6	Forward: 5ʹ-CTCGCTTCGGCAGCACA-3ʹ
	Reverse: 5ʹ-AACGCTTCACGAATTTGCGT-3ʹ
GAPDH	Forward: 5'-CACCCACTCCTCCACCTTTG-3'
	Reverse: 5ʹ-CCACCACCCTGTTGCTGTAG-3ʹ

*circCOL5A1* circular RNA COL5A1, *miR-370-5p* microRNA-370-5p, *PRKCSH* protein kinase C substrate 80K-H, *GAPDH* glyceraldehyde 3-phosphate dehydrogenase

### Cell culture

Human renal proximal tubular epithelial cell line (HK-2) and human RCC cell lines (786-O, KAKi-2, A498, ACHN, OS-RC-1, and OS-RC-2) were obtained from ATCC (VA, USA). RCC cell lines were cultured in RPMI 1640 medium supplemented with 1% streptomycin/penicillin and 10% FBS. HK-2 cells were cultured in DMEM (Gibco, China) with the same concentration of FBS and antibiotics. All cells were kept at 37 °C with 5% CO_2_.

### Actinomycin D and RNAse R analysis

ACHN cells were grown in 6-well plates (5 × 10^5^ cells/well) for 24 h, where 2 μg/ml actinomycin D (Sigma) was performed. At a specified time point, cells were harvested, and RT-qPCR was conducted to analyze RNA stability.

RNA (10 μg) from ACHN cells was mixed with RNAse R (3 U/g, Epicenter) at 37 °C for 30 min. circRNA and linear RNA were detected by RT-qPCR.

### Characteristic analysis of circCOL5A1

To confirm the authenticity of circCOL5A1 detection by RT-qPCR, amplified products were harvested. Agarose (2%) was prepared with Tris–acetate–EDTA buffer and fully dissolved by heating. Then, GelGreen (Biomed, Beijing, China) was added, and the mixture was solidified after 20 min. The amplified products were processed with gel electrophoresis and observed under ultraviolet light.

### Cell transfection

siRNA or pcDNA 3.1 overexpression vectors targeting circCOL5A1 and PRKCSH (si-circCOL5A1/PRKCSH, pcDNA 3.1-circCOL5A1/PRKCSH), miR-370-5p mimic/inhibitor, and their negative controls (si-NC, pcDNA 3.1, mimic/inhibitor NC) were supplied by GenePharma. At 70–80% confluence, ACHN cells were transfected with the above vectors as per the instruction of Lipofectamine 2000 (Invitrogen). RT-qPCR or Western blot was required to test gene expression, so as to verify successful transfection at 48 h.

### Colony counting

ACHN cells were cultured in 6-well plates (600 cells/well) for 2 weeks. The medium was changed every 3 days. On the day 14, cells fixed in 4% paraformaldehyde (Sigma-Aldrich) were stained with crystal violet solution (Sigma-Aldrich), washed with phosphate buffered saline, and allowed to air dry.

### EdU experiment

ACHN cells in the 96-well plates (1 × 10^4^ cells/well) were stained with EdU solution, Apollo567 solution, and DAPI solution according to the Cell-Light™ EdU Apollo567 in Vitro Imaging Kit (RiboBio). After treatments, cells were observed under a fluorescence microscope (ACCEXP Eclipse TI2-U, ACCEXP) and EdU-positive rates were analyzed using ImageJ software.

### Transwell assays

A 24-well Transwell chamber (Corning company) was utilized for the evaluation of migration and invasion. Matrigel (Corning) for invasion detection was covered in the upper chamber. The upper chamber contained cell suspensions and serum-free medium, and the lower one contained RPMI 1640 medium containing 10% FBS. Migrating or invading cells were immobilized with 4% paraformaldehyde and stained crystal violet (Sigma-Aldrich) at 24 h, and images were harvested with an inverted microscope (×100; Olympus).

### Flow cytometry

Concerning detecting ACHN cell apoptosis, the Annexin V/FITC Apoptosis assay kit (Southern Biotech) was utilized. ACHN cells after PBS cleaning (Invitrogen) were re-suspended in a binding buffer, mixed with 5 μL Annexin V-FITC/PI for 15 min, and loaded on the FACSan flow cytometer (BD Bioscience, Heidelberg, Germany) for apoptotic detection.

### Glycolysis assessment

The Glucose Absorption Test Kit (Biovision) measures glucose consumption. Lactic acid and ATP were measured by D-lactic acid detection kit and ATP colorimetric/fluorescent detection kit (Biovision), respectively. The corresponding absorbance was recorded on the microplate reader.

### FISH

ACHN cells fixed with 4% paraformaldehyde were permeated with 0.25%Triton X-100 in PBS for 15 min and treated with hybrid buffers (50% formamide, 10 ml Tris–HCl, pH 8.0, 200 μg/ml yeast tRNA, 1 × Denhardt solution, 600 ml NaCl, 0.25% SDS, 1 ml EDTA, and 10% dextran sulfate) for 1 h. After that, cells were incubated overnight at 37 °C in a hybrid buffer containing 50 nm biotin-labeled circCOL5A1 probe (Invitrogen) or 25 nm miR-370-5p probe (Invitrogen). After TBS washing, cells were reacted with TSA Cy5 kit (NEL745001KT) for 10 min and visualized with Prolong Gold anti-fading reagent containing DAPI. Images were captured using a microscope (Karl-Zeiss, LSM700).

### Western blot

After extracting proteins from tissues or cells based on RIPA lysis buffer (R0010, Solarbio), protein concentration was assessed with a BCA kit (Yeasen). Proteins were separated by electrophoresis of sodium dodecyl sulfate–polyacrylamide gel and then electrically transferred to a polyvinylidene fluoride membrane. Then, primary antibodies HK2 (22029-1-AP, Proteintech), PKM2 (4053, Cell Signaling Technology), PRKCSH (12148-1-AP, Proteintech), and GAPDH (ab8245, Abcam) were reacted at 4 °C overnight, and an HRP-labeled secondary antibody (1:20,000, Abcam) was re-treated for 1 h. After signal development using enhanced chemiluminescence, protein bands were quantified using Image J analysis software.

### Assessment of luciferase activity

circCOL5A1 and PRKCSH 3 'UTR containing miR-370-5p binding sequences and mutant sequences were produced and cloned into the pmirGLO–promoter vector (Promega). The products (wild type or mutant type pmirGLO–circCOL5A1/PRKCSH), along with miR-370-5p mimic or mimic NC, were transfected into ACHN cells following Lipofectamine 2000 (Invitrogen). The dual luciferase reporter assay system (Promega) measured luciferase activities at 48 h.

### RIP experiment

RIP buffers were configured with magnetic beads coupled with human anti-AgO2 antibody or mouse IgG. The buffer was mixed with the cell lysate to form a complex, which was digested with protease K to obtain immunoprecipitated RNA. After evaluations on the spectrophotometer (NanoDrop, Thermo Fisher Scientific), the purified RNA was tested by RT-qPCR.

### Xenotransplantation in nude mice

Ten 6-week-old male BALB/c nude mice (Vital River Laboratory Animal Technology, Beijing, China) were conditioned to a subcutaneous injection in the armpit with 1 × 10^6^ ACHN cells (after transfection with si-circCOL5A1 or si-NC). Based on weekly measurements of tumor size, tumor volume was calculated (length × width^2^/2). Tumors were harvested after 28 days, weighed, and processed for Western blot or IHC analysis [15]. The Animal Ethics Committee of The First Affiliated Hospital of Shaoyang University approved the animal study.

### Data analysis

Statistical analysis was performed using GraphPad Prism 9.0 software. All experiments were biologically replicated at least three times. Shown as mean ± standard deviation (SD), data were compared by one-way ANOVA and Tukey multiple comparison tests (≥ 3 groups) or unpaired student *t* test (2 groups). Whether circCOL5A1 expression was associated with clinical features in RCC patients was determined by Chi-square test. *P* < 0.05 was considered a significant difference.

## Results

### circCOL5A1 high expression in RCC

**Fig. 1:**
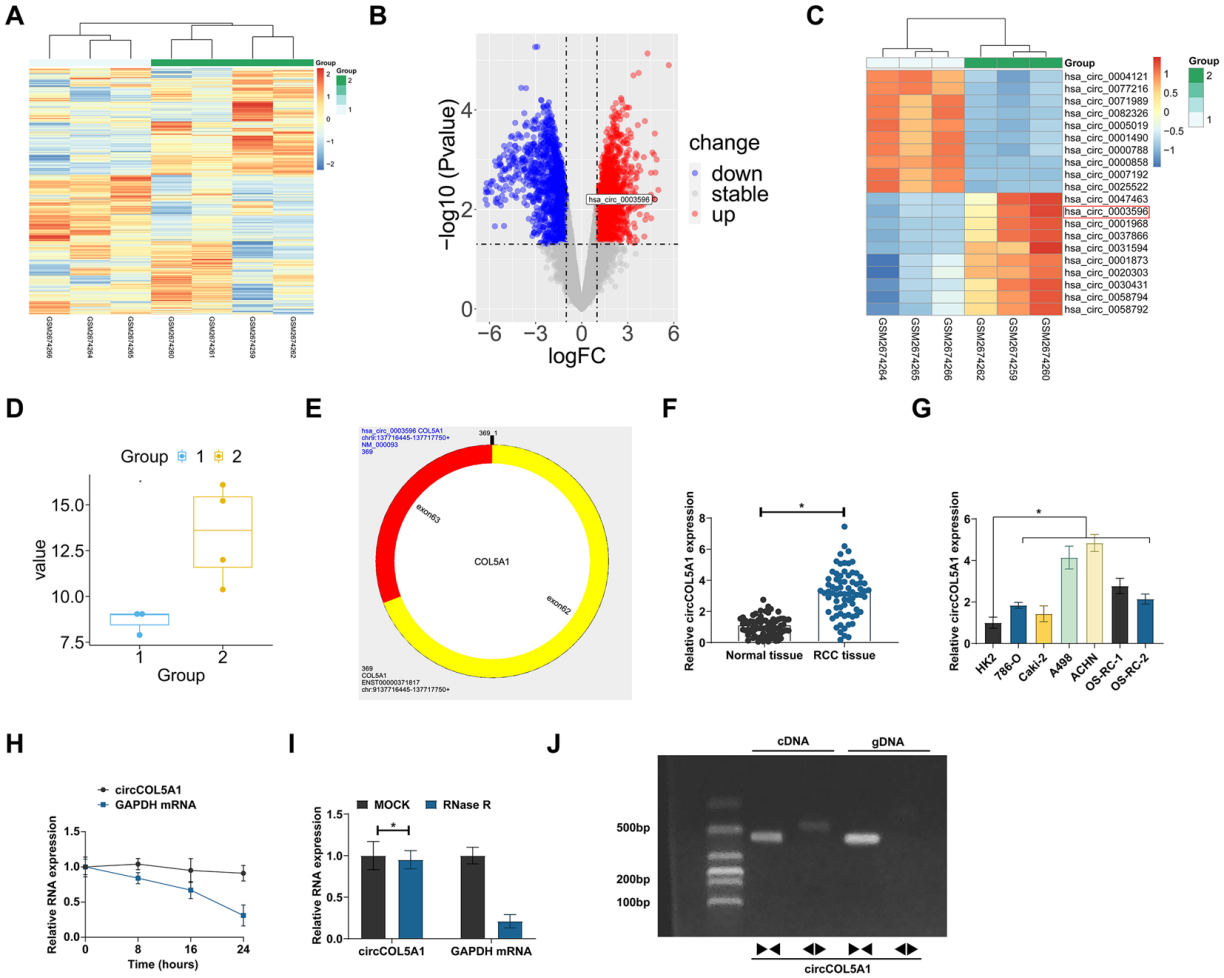
Abnormally high expression of circCOL5A1 in RCC. **A** Heat map of differential expression of circRNAs in normal tissue and RCC tissue; **B** volcanic maps of different circRNAs; **C** top 10 circRNAs with highest expression and lowest expression; **D** changes of circCOL5A1 expression; **E** genetic information of circCOL5A1; **F** circCOL5A1 in RCC tissues and normal tissues analyzed by RT-qPCR. **G** circCOL5A1 in HK-2 and human RCC cell lines analyzed by RT-qPCR; **H**, **I** the stability of circCOL5A1 evaluated by actinomycin D assay and RNAse R experiment; **J** gel electrophoresis to examine the products of RT-qPCR amplification using polymeric and diverging primers; data were expressed as mean ± SD (*N* = 3). * *P* < 0.05

In 3 pairs of paracancer normal tissue and RCC tissue from the bioinformatics website http://www.biomedical-web.com, circRNAs were analyzed (Fig. 1A). Among the first 10 abnormally and highly expressed circRNAs, hsa_circ_0003596 was a circRNA of interest and named circCOL5A1 (the first four highly expressed circRNAs were not selected, because they were not detected with high expression in some RCC cell lines) (Fig. 1B, C). According to circbase query, circCOL5A1 is located on exons 62 and 63 of the COL5A1 gene, with a length of 369 bp, and its difference change multiple is 4.7644 (Fig. 1D, E). As measured, circCOL5A1 in RCC tissues and 6 RCC cell lines was higher than that in the normal control (Fig. 1F, G). circCOL5A1 ring structure was then analyzed. circCOL5A1 had a worse half-life than GAPDH mRNA after actinomycin D treatment (Fig. 1H). RNAse R degraded linear GAPDH mRNA, but not circCOL5A1 (Fig. 1I). To further confirm the characteristics of circCOL5A1, specific primers were designed to amplify linear and circular COL5A1 sequences. RT-qPCR analysis confirmed that circCOL5A1 could only be amplified from cDNA templates using diverging primers, while linear COL5A1 mRNA could be detected from both cDNA and gDNA templates using convergent primers (Fig. 1J). These data suggest that circCOL5A1, as a novel circRNA, is highly expressed in RCC and may be involved in RCC development.

### circCOL5A1 knockdown inhibited RCC proliferation, invasion and migration, and glycolysis

**Fig. 2:**
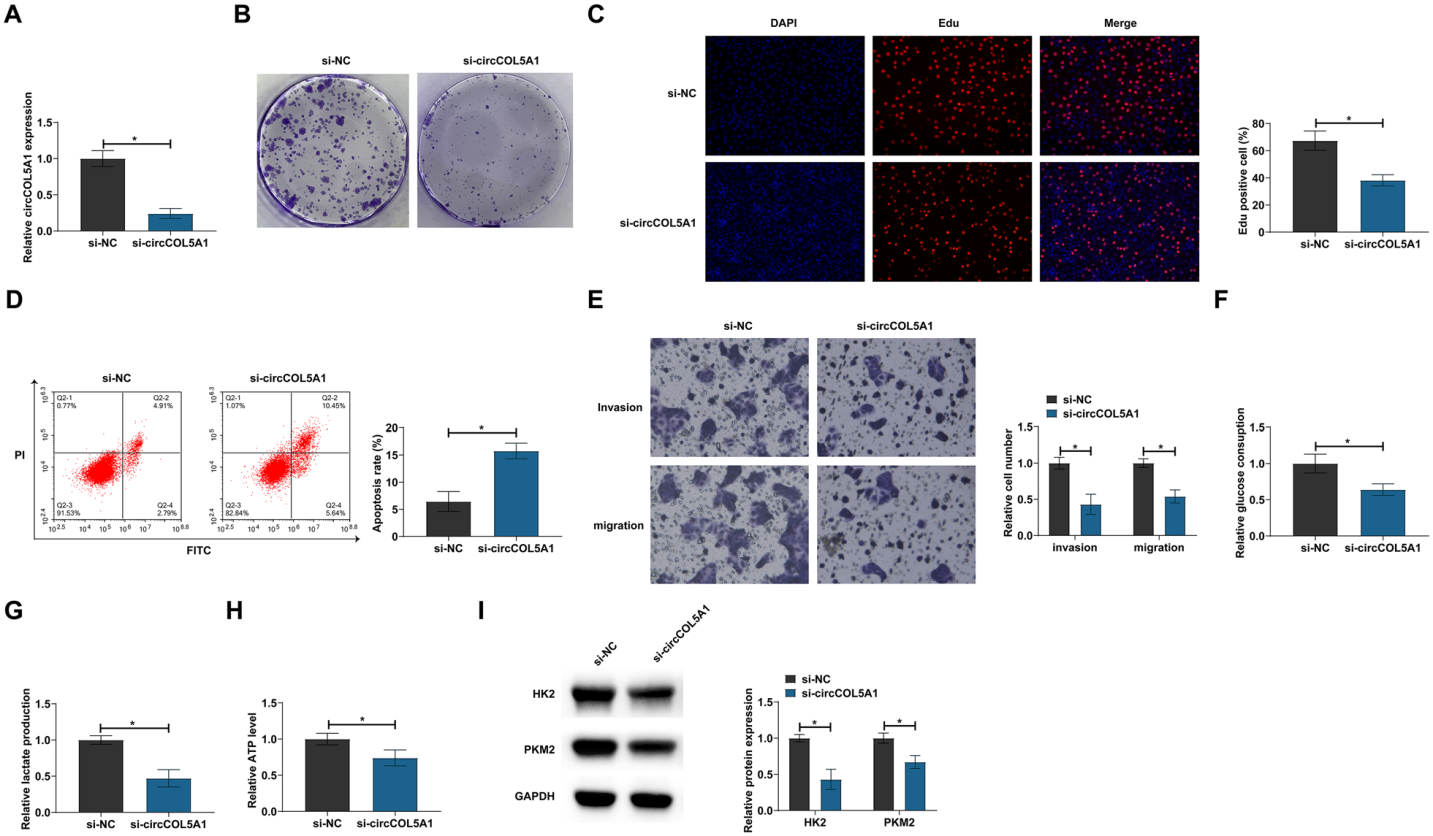
circCOL5A1 knockdown impairs RCC progression. circCOL5A1 targeted siRNA was transfected into ACHN cells. **A** circCOL5A1 expression analyzed by RT-qPCR; **B**, **C** cell proliferation detected by clonal assay and EdU assay; **D** apoptosis evaluated by flow cytometry; **E** invasion and migration evaluated by Transwell assays; **F**–**H** glucose consumption, lactic acid production, and ATP levels; **I** HK2 and PKM2 detected by Western blot; data were expressed as mean ± SD (*N* = 3). * *P* < 0.05

circCOL5A1-targeted siRNA was introduced into ACHN cells. Successful knockdown of circCOL5A1 was confirmed by RT-qPCR (Fig. 2A). Cell proliferation was analyzed by clonogenic assay and EdU proliferation assay. Knocking down circCOL5A1 reduced the number of cloned cells and the EdU-positive rate (Fig. 2B, C). Apoptosis was then assessed by flow cytometry, whereas invasion and migration were by Transwell assays. Suppressing circCOL5A1 increased the apoptosis rate (Fig. 2D) and weakened invasive and migratory capacities (Fig. 2E). Glycolysis provides great help to the malignant behavior of cancer [16]. Therefore, the research subsequently investigated whether circCOL5A1 is related to the glycolysis of RCC. Data demonstrated that circCOL5A1 knockdown reduced glucose consumption, lactate production, and ATP levels (Fig. 2F–H), as well as inhibited protein expression of glycolytic-related proteins (HK2 and PKM2) (Fig. 2I). These data suggest that knockdown of circCOL5A1 can inhibit RCC cell proliferation, invasion, migration, and glycolysis.

### circCOL5A1 competitively adsorbs miR-370-5p

**Fig. 3:**
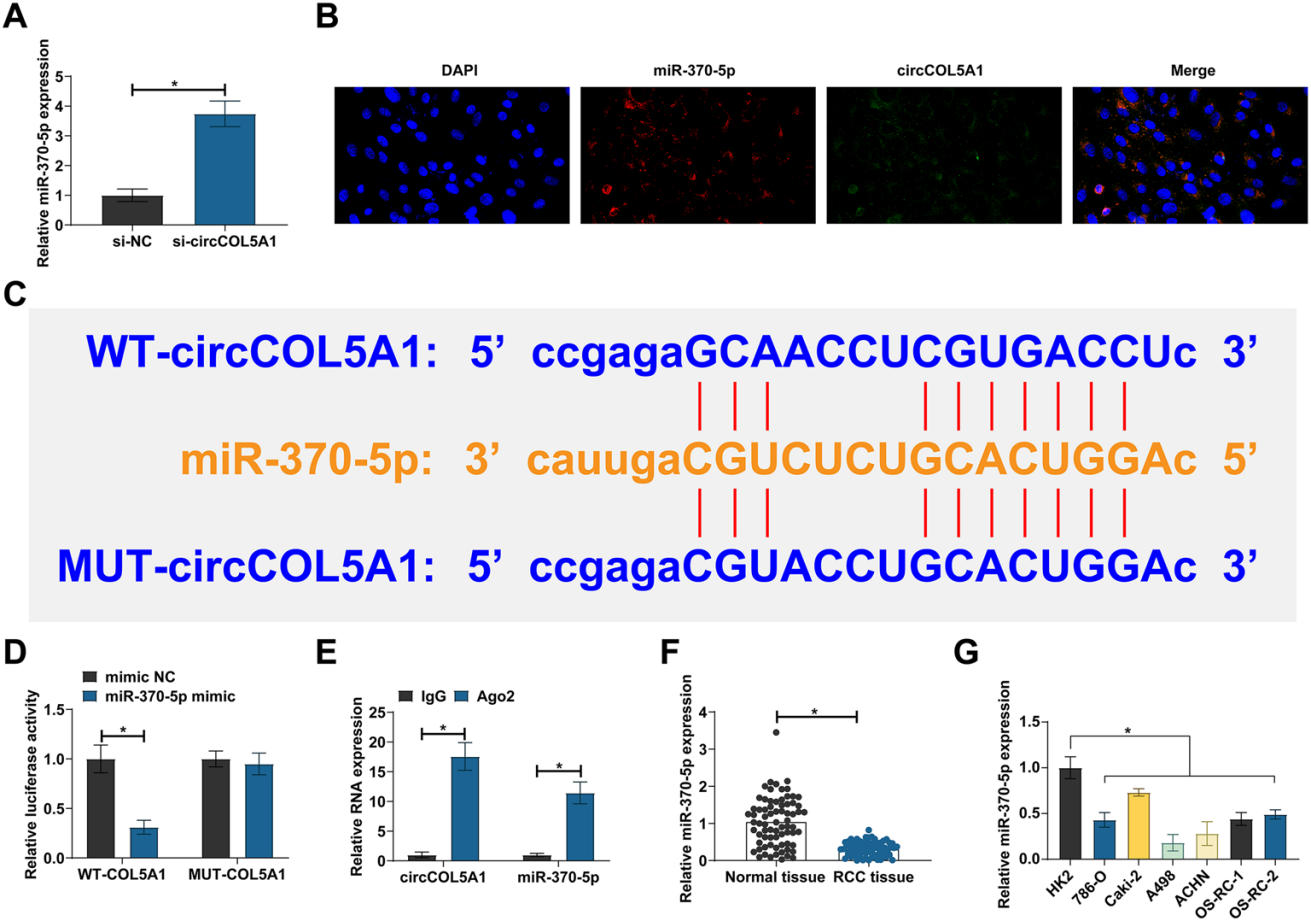
Competitive adsorption of miR-370-5p by circCOL5A1 in RCC. **A** RT-qPCR detection of miRNA expression after circCOL5A1 knockdown; **B** subcellular localization of miR-370-5p and circCOL5A1 assayed by FISH; **C** potential binding sites of miR-370-5p and circCOL5A1; **D**, **E** the targeting relationship between miR-370-5p and circCOL5A1 detected by dual luciferase reporter assay and RIP experiment; **F** miR-370-5p expression in RCC tissues and normal tissues analyzed by RT-qPCR; G: miR-370-5p expression in HK-2 and human RCC cell lines analyzed by RT-qPCR; data were expressed as mean ± SD (*N* = 3). * *P* < 0.05

Five miRNAs with potential binding sites to circCOL5A1 were selected from the bioinformatics website starbase. RT-qPCR showed that only miR-370-5p was negatively regulated by circCOL5A1 in ACHN cells (Fig. 3A). Subsequently, the subcellular localization of miR-370-5p and circCOL5A1 was examined by FISH assay. Both miR-370-5p and circCOL5A1 were located in the cytoplasm of ACHN cells (Fig. 3B). WT/MUT–circCOL5A1 luciferase reporter vectors were designed based on the predicted binding sites (Fig. 3C). Dual luciferase assay showed that co-transfection of WT–circCOL5A1 and miR-370-5p mimic could reduce luciferase activity (Fig. 3D). RIP tested the enrichment of circCOL5A1 and miR-370-5p in Ago2 magnetic beads (Fig. 3E). Meanwhile, RT-qPCR detected lower miR-370-5p expression in RCC tissues and cell lines than normal controls (Fig. 3F, G). These data indicate that circCOL5A1 competitively adsorbs miR-370-5p.

### miR-370-5p is involved in the process of circCOL5A1 regulating RCC

**Fig. 4:**
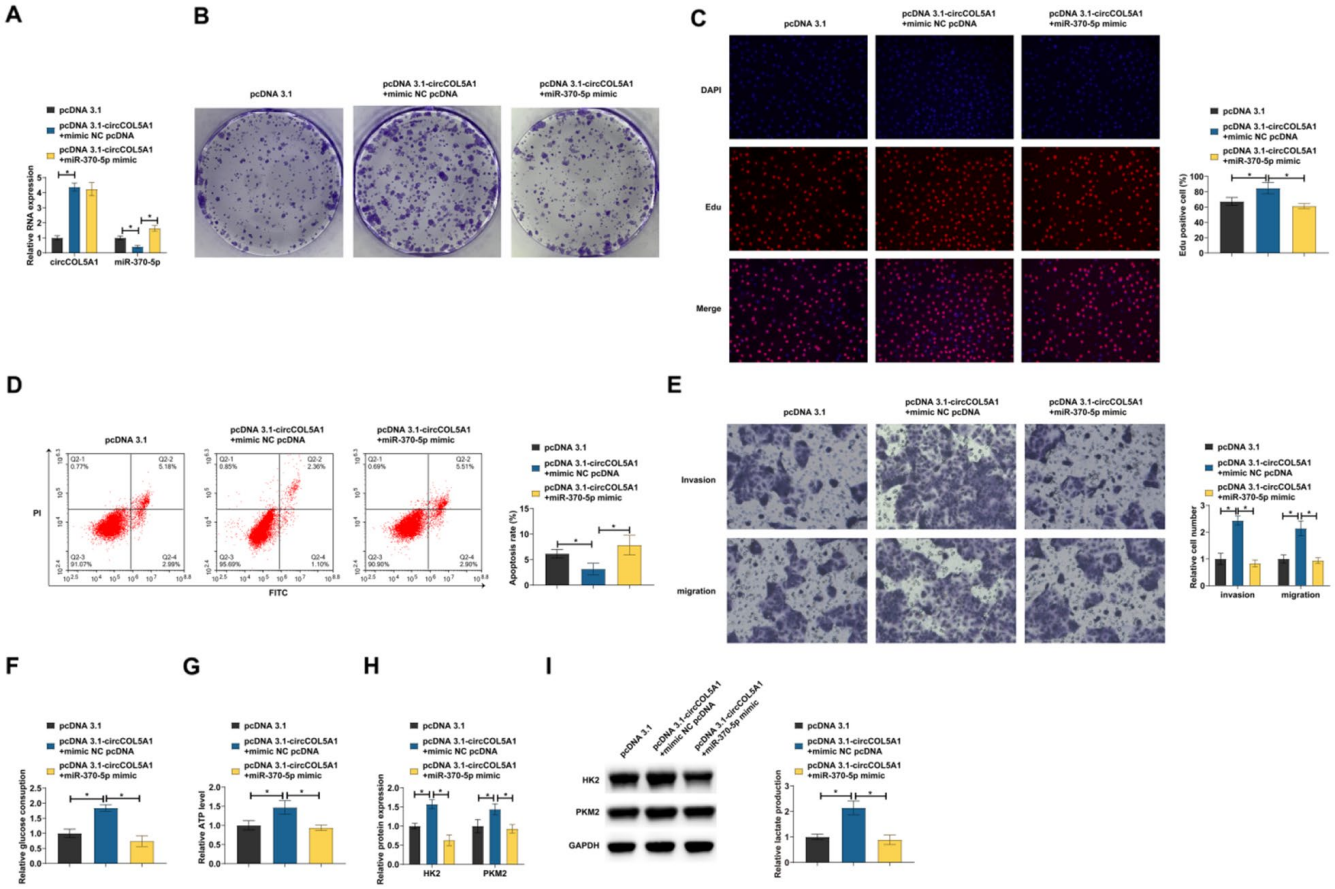
miR-370-5p is involved in the process of circCOL5A1 regulation of RCC. pcDNA 3.1-circCOL5A1 and miR-370-5p mimic were co-transfected into ACHN cells. **A** circCOL5A1 and miR-370-5p expression analyzed by RT-qPCR; **B**, **C** cell proliferation detected by clonal assay and EdU assay; **D** apoptosis evaluated by flow cytometry; **E** invasion and migration evaluated by Transwell assays; **F**–**H** glucose consumption, lactic acid production, and ATP levels; **I** HK2 and PKM2 detected by Western blot; Data were expressed as mean ± SD (*N* = 3). * *P* < 0.05

Functional rescue experiments were conducted to investigate whether miR-370-5p was involved in the regulation of RCC by circCOL5A1. pcDNA 3.1-circCOL5A1 and miR-370-5p mimic were co-transfected into ACHN cells. pcDNA 3.1-circCOL5A1 increased circCOL5A1 and reduced miR-370-5p, but miR-370-5p mimic restored miR-370-5p levels (Fig. 4A). pcDNA 3.1-circCOL5A1 increased the number of clone cells and EdU-positive rate, reduced the apoptosis rate, stimulated invasion and migration, and increased glucose consumption, lactic acid production, and ATP levels in cells. However, these impacts were counteracted by miR-370-5p mimic (Fig. 4B–I).

### miR-370-5p is targeted to regulate PRKCSH

**Fig. 5:**
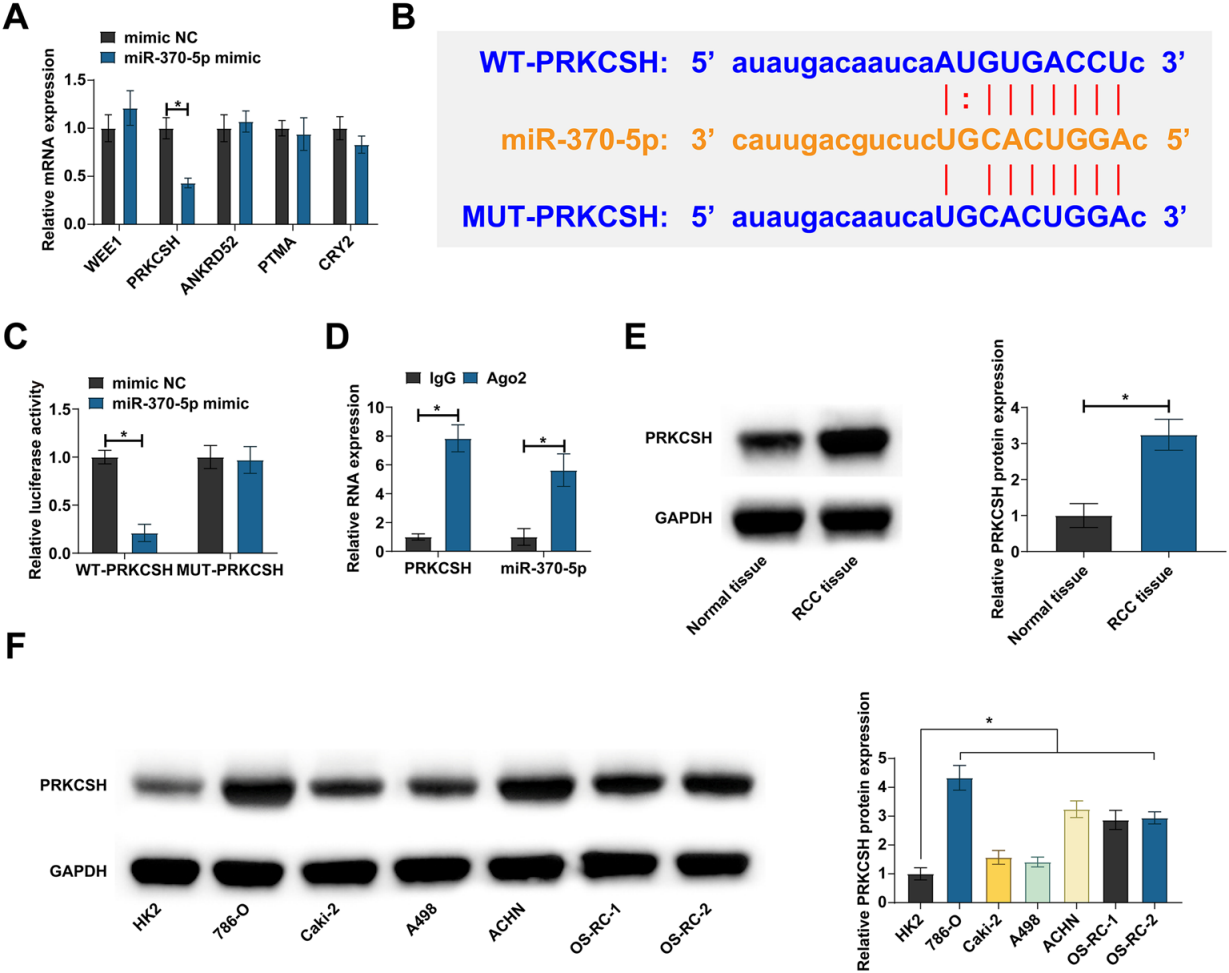
Targeted regulation of PRKCSH by miR-370-5p. **A** RT-qPCR detection of mRNA expression after overexpressing miR-370-5p; **B** potential binding sites of miR-370-5p and PRKCSH; **C**, **D** the targeting relationship between miR-370-5p and PRKCSH detected by dual luciferase reporter assay and RIP experiment; **E** PRKCSH expression in RCC tissues and normal tissues analyzed by Western blot; **F** PRKCSH expression in HK-2 and human RCC cell lines analyzed by Western blot; data were expressed as mean ± SD (*N* = 3). * *P* < 0.05

Five mRNAs with potential binding sites to miR-370-5p were selected by starbase. By RT-qPCR screening, PRKCSH was found to be negatively regulated by miR-370-5p in ACHN cells (Fig. 5A). Subsequently, their targeting relationship was analyzed. Co-transfection of WT-PRKCSH and miR-370-5p mimic reduced luciferase activity, and significant enrichment of them was found in Ago2 beads (Fig. 5B–D). Then, PRKCSH expression pattern in RCC was analyzed. Western blot showed that PRKCSH was highly expressed in both RCC tissues and cell lines (Fig. 5E, F). These data indicate that PRKCSH is a downstream target gene of miR-370-5p.

### *circCOL5A1 affects RCC progression *via* miR-370-5p/PRKCSH axis*

**Fig. 6:**
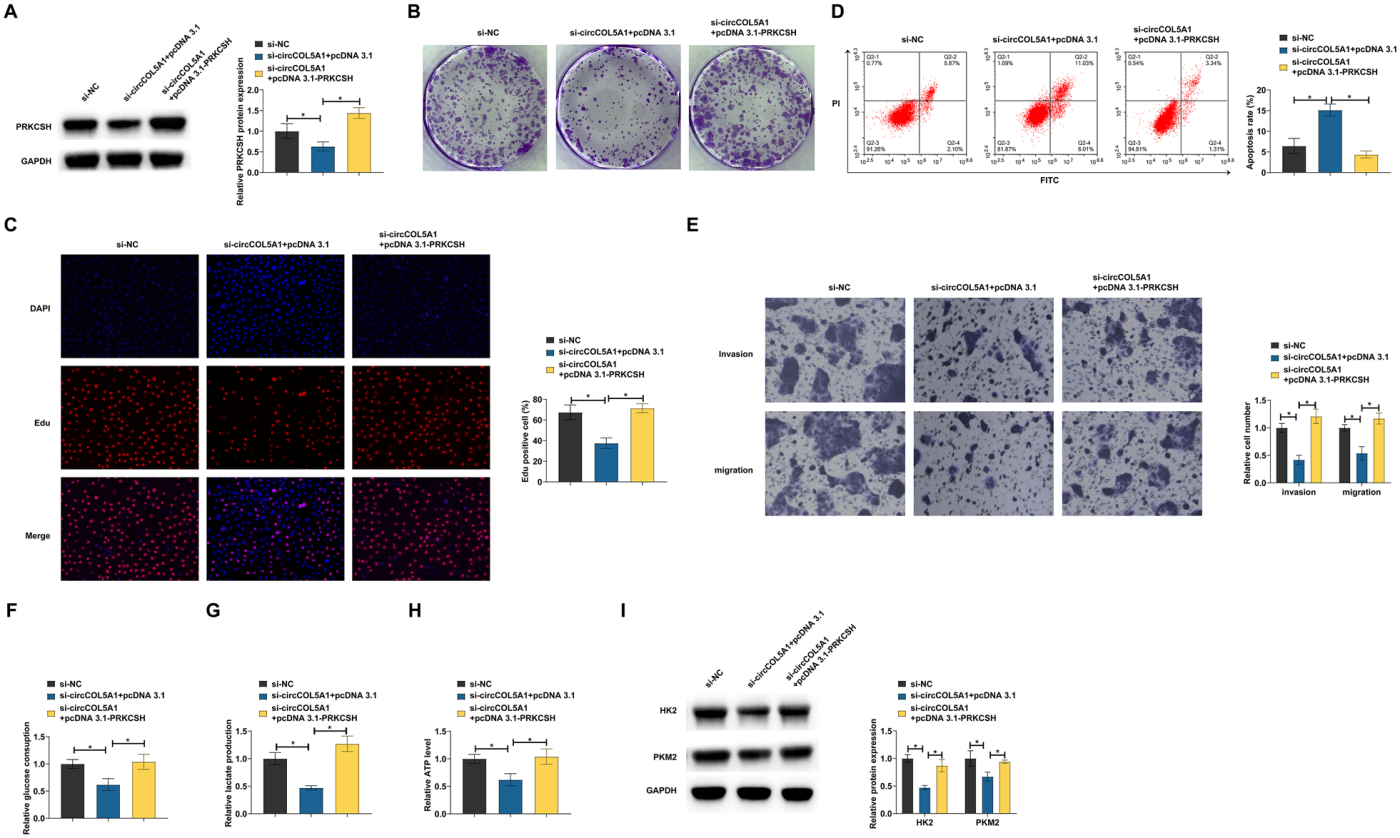
CircCOL5A1 affects RCC progression via miR-370-5p/PRKCSH axis. si-circCOL5A1 and pcDNA 3.1-PRKCSH were co-transfected into ACHN cells. **A** PRKCSH expression analyzed by Western blot; **B**, **C** cell proliferation detected by clonal assay and EdU assay; **D** apoptosis evaluated by flow cytometry; **E** invasion and migration evaluated by Transwell assays; **F**–**H** glucose consumption, lactic acid production, and ATP levels; **I** HK2 and PKM2 detected by Western blot; Data were expressed as mean ± SD (*N* = 3). * *P* < 0.05

Whether PRKCSH is a downstream functional gene of circCOL5A1/miR-370-5p was evaluated by functional rescue experiments. circCOL5A1-targeted siRNA and pcDNA 3.1-PRKCSH were co-transfected into ACHN cells. si-circCOL5A1 inhibited PRKCSH expression, but this effect was rescued by pcDNA 3.1-PRKCSH (Fig. 6A). circCOL5A1 knockdown decreased the number of cloned cells and EdU positive rate, increased apoptosis rate, inhibited invasion and migration ability, reduced glucose consumption, lactic acid production, and ATP levels, and decreased HK2 and PKM2 protein expression, but these effects were reversed by pcDNA 3.1-PRKCSH (Fig. 6B–I). These data suggest that circCOL5A1 promotes RCC proliferation, invasion and migration, and glycolysis by regulating the miR-370-5p/PRKCSH axis.

### CircCOL5A1 silencing delays the growth of RCC tumors

**Fig. 7:**
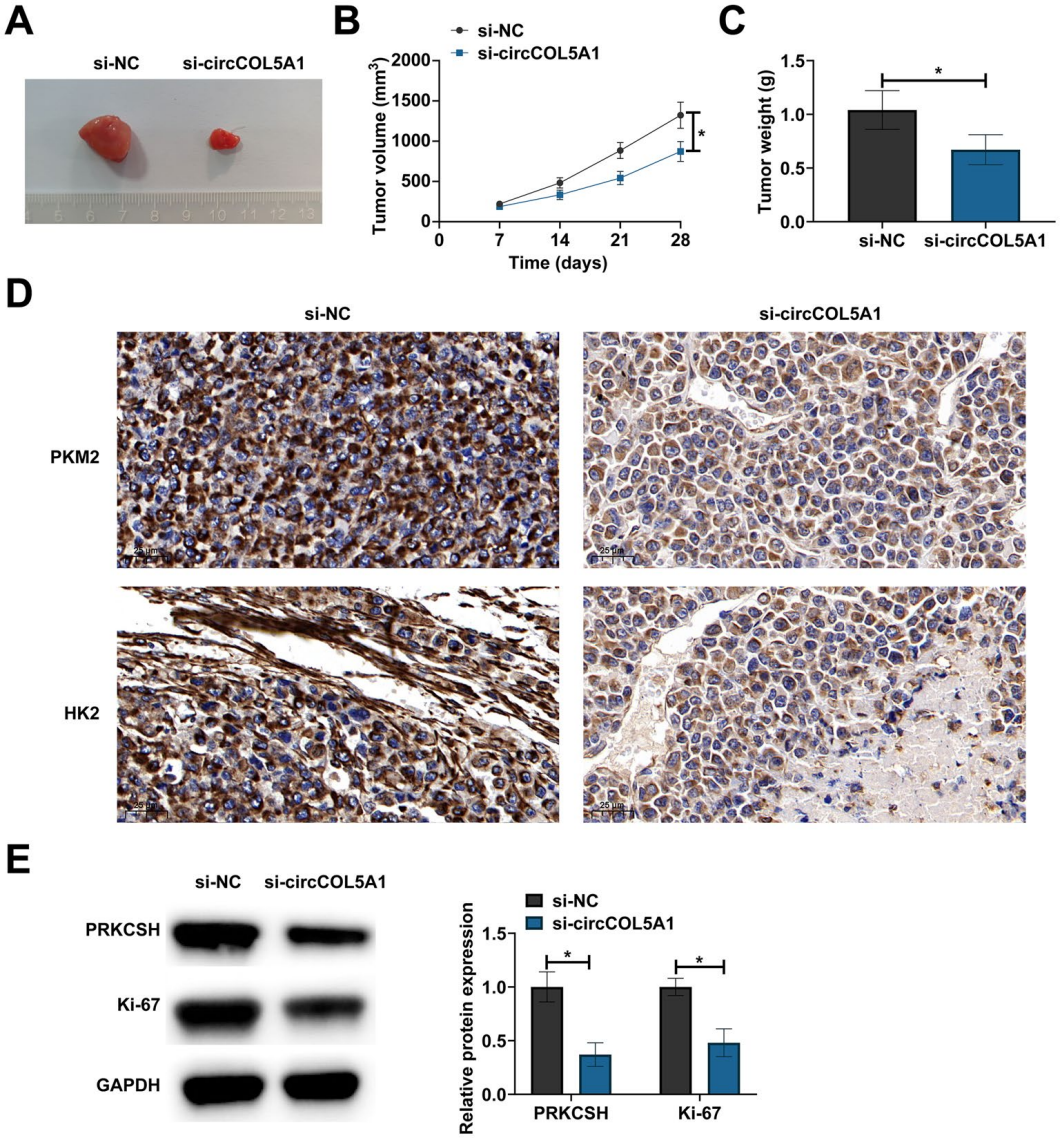
CircCOL5A1 silencing delays the growth of RCC tumors. **A** representative pictures of tumors; **B**, **C** tumor volume and weight; **D** HK2 and PKM2 in tumors detected by IHC staining; **E** PRKCSH and Ki-67 in tumors detected by Western blot. Data were expressed as mean ± SD (*n* = 5). * *P* < 0.05

The research investigated the effect of circCOL5A1 on the growth of RCC tumors. circCOL5A1 knockdown reduced tumor volume and weight (Fig. 7A–C). IHC staining observed lower HK2 and PKM2 expression in tumors after circCOL5A1 knockdown (Fig. 7D), and Western blot assessed lower protein expression of tumor PRKCSH and Ki-67 (Fig. 7E). Simply put, knocking down circCOL5A1 inhibits RCC tumor growth and glycolysis.

## Discussion

RCC is an overwhelming renal tumor, with a high rate of metastasis and a poor prognosis [17]. Due to metabolic reprogramming, cancer cells exhibit high glycolysis rates, resulting in lactate overproduction and increased extracellular acidity [18]. In addition, tumor glycolysis and lactate production affect the tumor immune microenvironment through metabolic competition with infiltrating immune cells [19]. Therefore, targeting glycolysis has become a promising direction for tumor therapy. Specifically, this study focused on and discussed the action of circCOL5A1 in controlling glycolysis of RCC cells, and the collected data presented that circCOL5A1, as a novel oncogene, encourages the malignant behavior of RCC by promoting cellular glycolysis, which is potentially related to the axis of miR-370-5p/PRKCSH.

Even when oxygen is plentiful, most of the glucose in cancer cells is converted to lactic acid, and this reprogramming of glucose metabolism has now been identified as a key hallmark of cancer [20]. More and more studies have focused on circRNAs in the aerobic glycolysis of RCC. circRNA influences the transcription, protein stability and enzyme activity of key enzymes and transporters of RCC glycolysis by regulating downstream target genes, and participates in glucose metabolism. CircME1 and circ_0008717 promote glycolysis in clear cell RCC [21, 22]. This study discovered a new circRNA (hsa_circ_0003596, circCOL5A). circCOL5A expression was abnormally high in RCC, and circCOL5A knockdown effectively inhibited migration behaviors of RCC cells. In addition, knocking down circCOL5A effectively reduced lactic acid production and glucose consumption in RCC cells. Because in RCC cells, glycolysis allows RCC cells to metabolize glucose efficiently despite hypoxia and produce enough energy to support their unusually rapid proliferation [23]. This metabolic alteration allows RCC cells to direct metabolites to biosynthetic pathways, such as nucleotide and fatty acid biosynthesis, which are the raw materials necessary for cell proliferation [24]. In addition, the accumulation of lactic acid during glycolysis can also promote cellular oxidative stress, thus stimulating the proliferation and survival of RCC cells [25]. The production of lactic acid acidifies the tumor microenvironment, which helps to reduce the adhesion of the extracellular matrix, thus making it easier for RCC cells to invade surrounding tissues [26]. Therefore, we believe that circCOL5A accelerates RCC proliferation, invasion, and migration mainly by promoting glycolysis.

Studies have confirmed that circRNA is involved in regulating the immune escape process of RCC. For example, circAGAP1 can stimulate immune escape and distant metastasis of RCC [27]. During glycolysis, the accumulation of lactic acid will reduce the activity of CD8^+^ T cells (cytotoxic T cells), and inhibit the cytotoxicity and cytolysis ability of other immune cells, thus weakening the attack effect on RCC cells [28]. In addition, RCC cells acquire large amounts of glucose through highly active glycolysis and direct it to the biosynthetic pathway, thereby limiting glucose supply to immune cells [29, 30]. These effects can cause immune cells to have difficulty surviving in the tumor microenvironment, thus promoting the immune escape process. circCOL5A can promote glucose consumption and increase lactic acid production in RCC cells, so we speculate that circCOL5A also promotes RCC immune escape, which needs to be explored in future studies.

It is well-established that many ncRNAs act as nodes or hubs in the ceRNA network, and disruptions in their interactions can lead to the development of cancer. Given that, downstream miRNAs of circCOL5A1 received great attention in RCC, and miR-370-5p eventually became the study focus. In proceeding studies, miR-370-5p is inclined to control tumor development as a tumor suppressor. As an example, Sang et al. have observed the downregulation of miR-370-5p in breast cancer, and have experimentally confirmed that miR-370-5p overexpression represses proliferation and invasion of cancer cells. Intriguingly, miR-370-5p can obstruct bladder cancer cells to proliferate, migrate, and invade [14]. Moreover, downregulating miR-370-5p induces proliferative and invasive actions of colorectal carcinoma cells [31]. In the course of RCC, the current study detected miR-370-5p expression at a low level, and artificial upregulation of miR-370-5p saved the pro-tumor effect of circCOL5A1 on RCC cells, as evidenced by suppression of tumor malignancy and glycolysis.

mRNAs are significant parts of ceRNA networks due to miRNA targeting. The current research kept eyes on PRKCSH, the target of miR-370-5p in RCC. Notably, Sudo M et al. have obtained a recognition that PRKCSH silencing induces apoptotic activity of non-small cell lung cancer after gefitinib treatment [32]. To our best knowledge, PRKCSH expression is higher in lung cancer, showing a correlation with patients’ prognosis, and loss of PRKCSH arrests cells in the G2/M phase after zinc oxide nanoparticle therapy [33]. Consistently, PRKCSH expression was detected to be elevated in RCC, and PRKCSH overexpression contributed to a reversal of the anti-tumor effect of circCOL5A1 silencing.

In terms of study limitation, the effect of miR-370-5p and PRKCSH should be analyzed in animal experiments. In addition, the sample size needs to be expanded to further support the obtained results. Finally, other molecules mediated by circCOL5A1 and potential signaling pathways become further study focuses. All in all, this research summarizes the tumor-promoting effect of circCOL5A1 in the field of glycolysis by competing with miR-370-5p to release PRKCSH expression (Additional file 1).

### Supplementary Information

**Additional file 1. **Protein bands in Western blot.

## Data Availability

The data sets used and/or analyzed during the present study are available from the corresponding author on reasonable request.
