# Supplementary figures and images for: hsa_circ_0003596, as a novel oncogene, regulates the malignant behavior of renal cell carcinoma by modulating glycolysis
Source: Eur J Med Res. 2023 Sep 2;28:315. doi: 10.1186/s40001-023-01288-z (PMC10474667; doi:10.1186/s40001-023-01288-z)

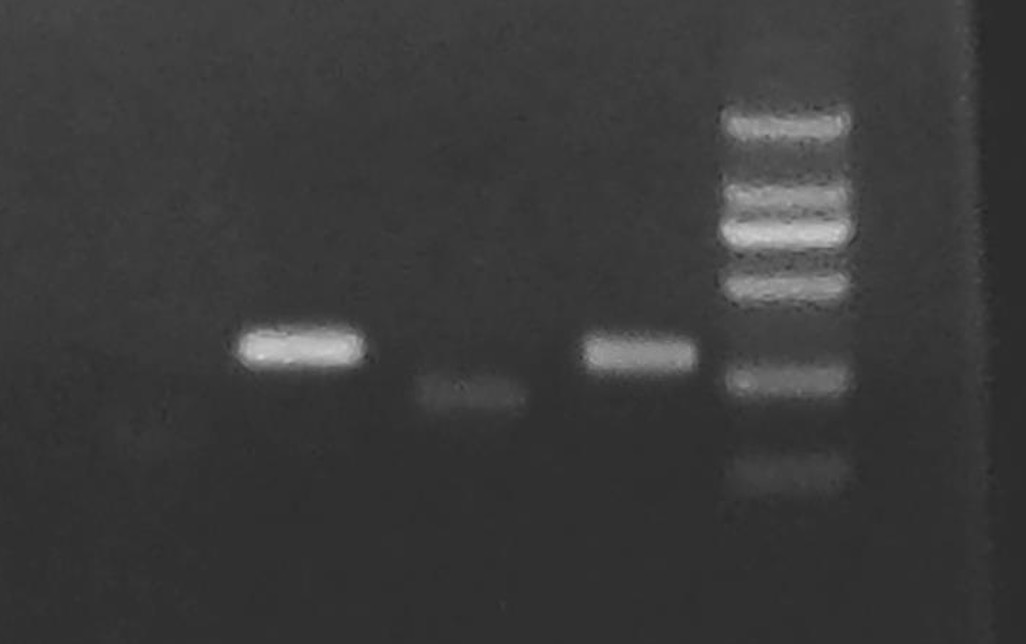

Supplement: Supplementary file 1 — Additional file 1. Protein bands in Western blot. [file 40001_2023_1288_MOESM1_ESM.zip › original image for western blot/Figure 1J.jpg]

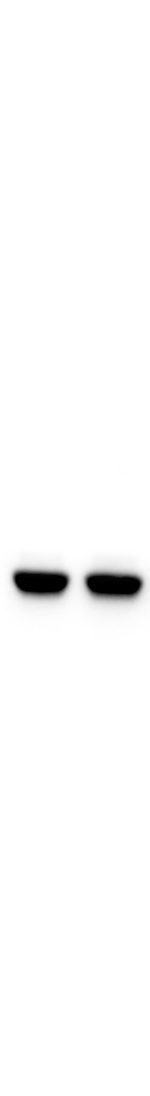

Supplement: Supplementary file 1 — Additional file 1. Protein bands in Western blot. [file 40001_2023_1288_MOESM1_ESM.zip › original image for western blot/Figure 2I/GAPDH.JPG]

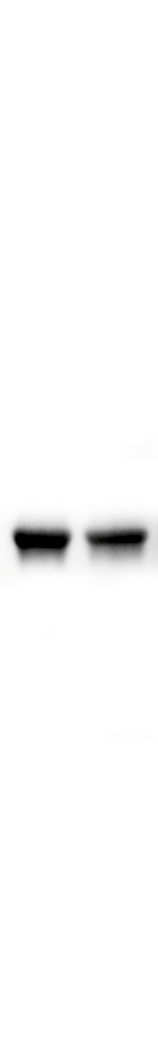

Supplement: Supplementary file 1 — Additional file 1. Protein bands in Western blot. [file 40001_2023_1288_MOESM1_ESM.zip › original image for western blot/Figure 2I/HK2.jpg]

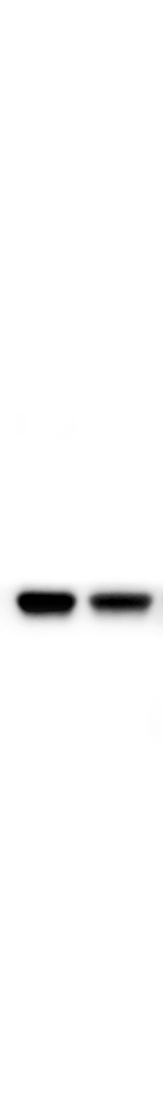

Supplement: Supplementary file 1 — Additional file 1. Protein bands in Western blot. [file 40001_2023_1288_MOESM1_ESM.zip › original image for western blot/Figure 2I/PKM2.jpg]

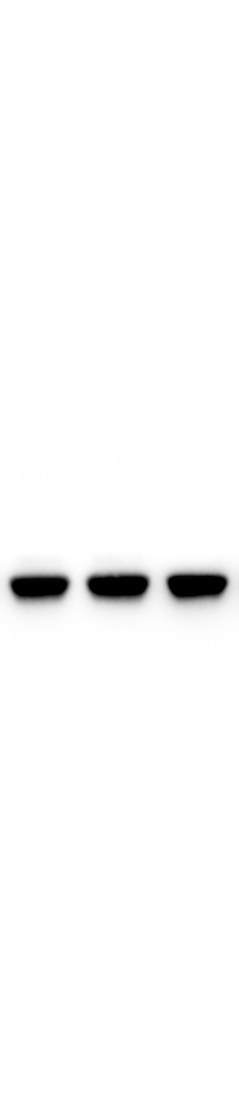

Supplement: Supplementary file 1 — Additional file 1. Protein bands in Western blot. [file 40001_2023_1288_MOESM1_ESM.zip › original image for western blot/Figure 4I/GAPDH.JPG]

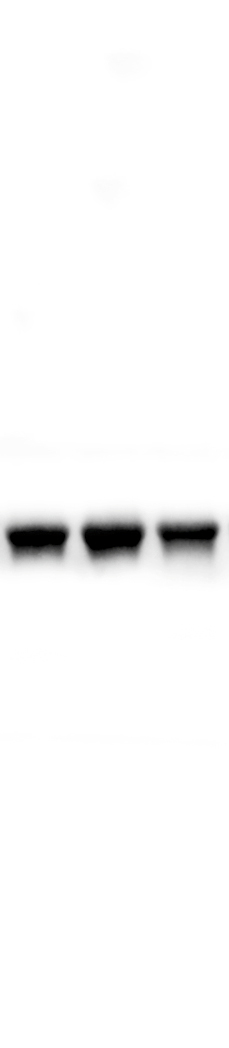

Supplement: Supplementary file 1 — Additional file 1. Protein bands in Western blot. [file 40001_2023_1288_MOESM1_ESM.zip › original image for western blot/Figure 4I/HK2.jpg]

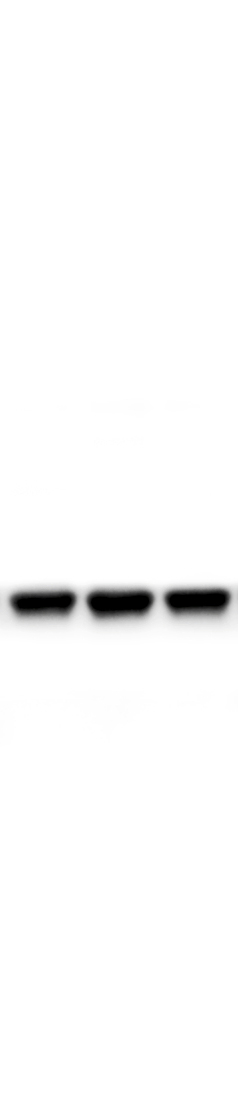

Supplement: Supplementary file 1 — Additional file 1. Protein bands in Western blot. [file 40001_2023_1288_MOESM1_ESM.zip › original image for western blot/Figure 4I/PKM2.jpg]

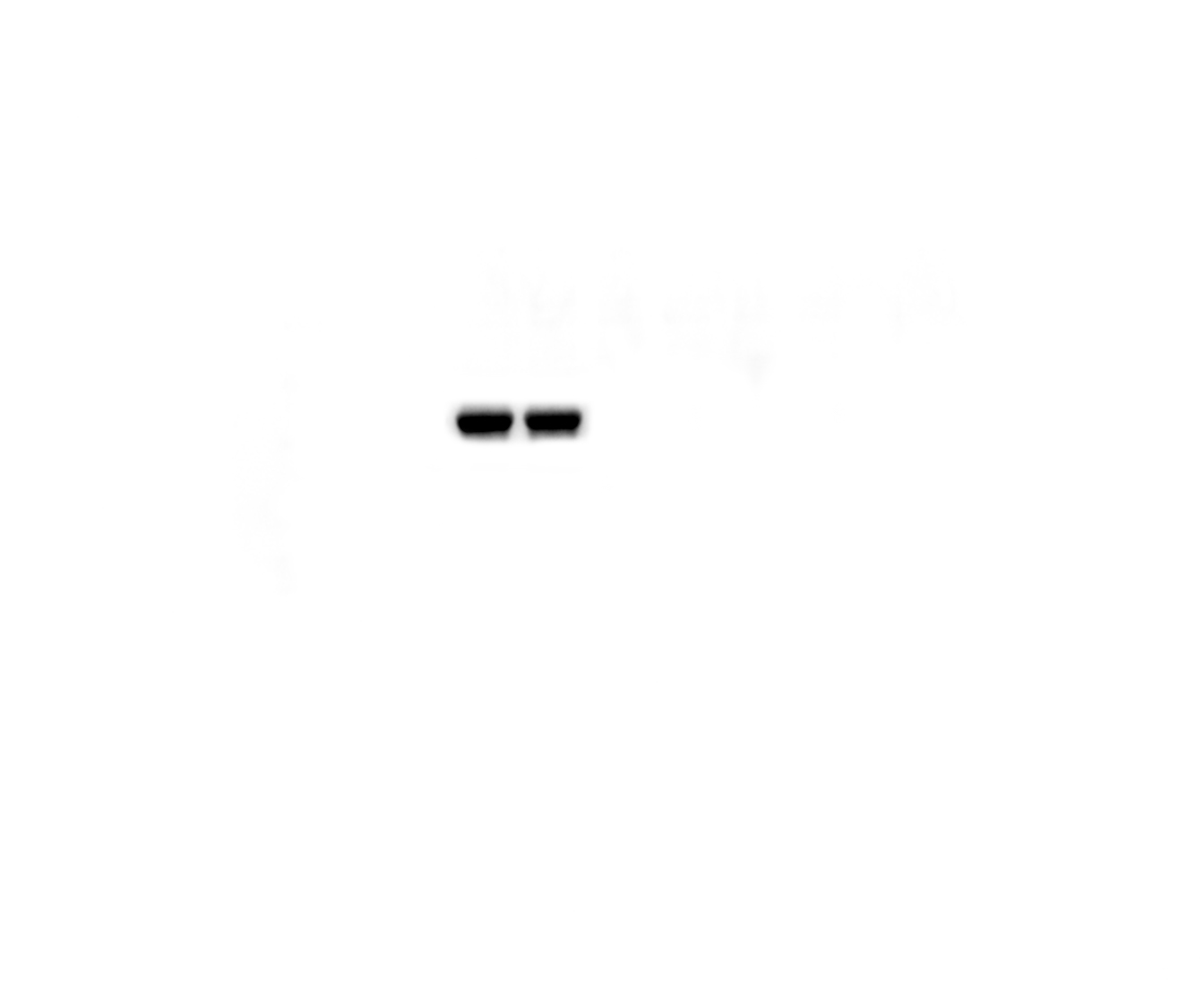

Supplement: Supplementary file 1 — Additional file 1. Protein bands in Western blot. [file 40001_2023_1288_MOESM1_ESM.zip › original image for western blot/Figure 5E/GAPDH.jpg]

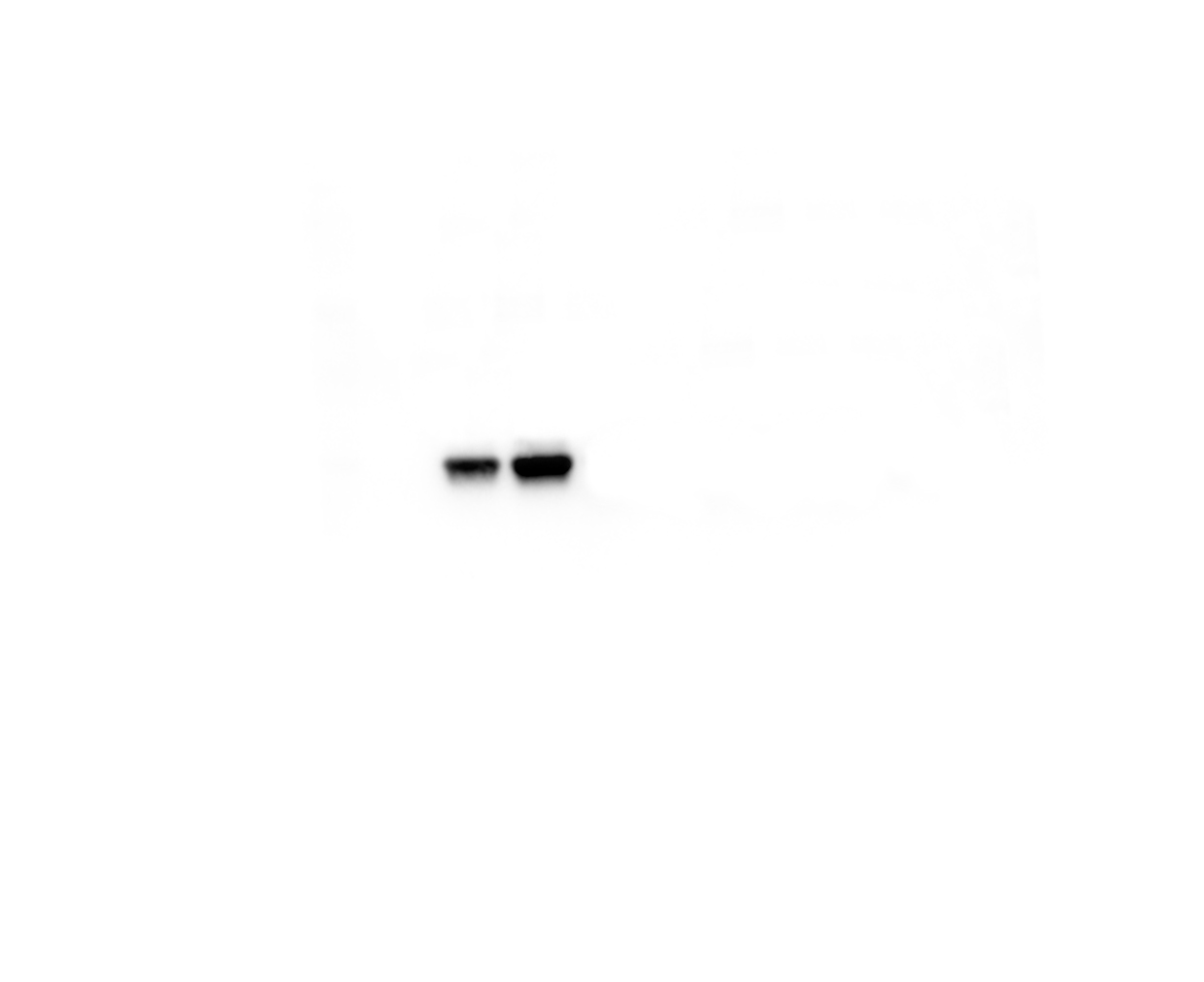

Supplement: Supplementary file 1 — Additional file 1. Protein bands in Western blot. [file 40001_2023_1288_MOESM1_ESM.zip › original image for western blot/Figure 5E/PRKCSH.jpg]

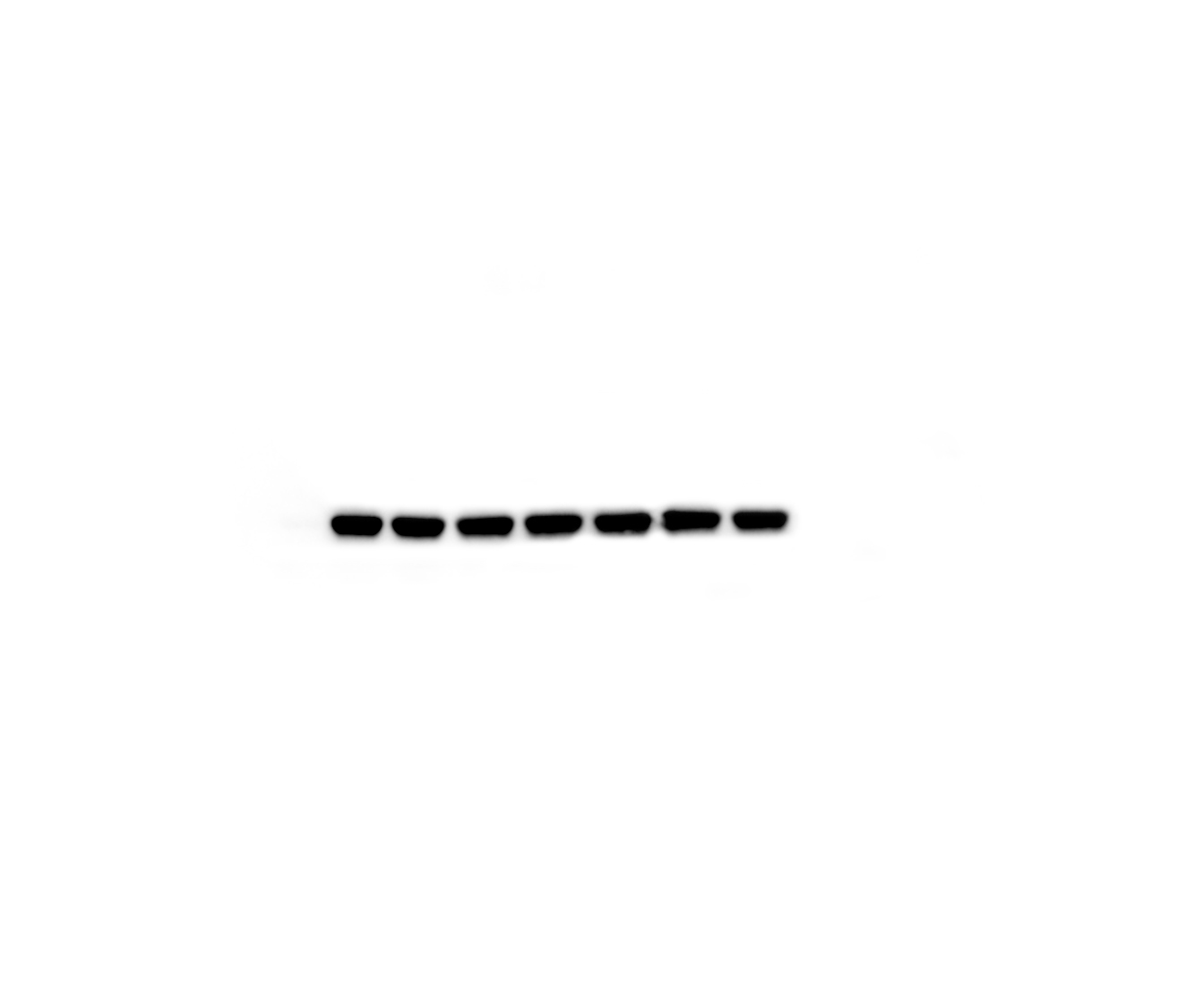

Supplement: Supplementary file 1 — Additional file 1. Protein bands in Western blot. [file 40001_2023_1288_MOESM1_ESM.zip › original image for western blot/Figure 5F/GAPDH.jpg]

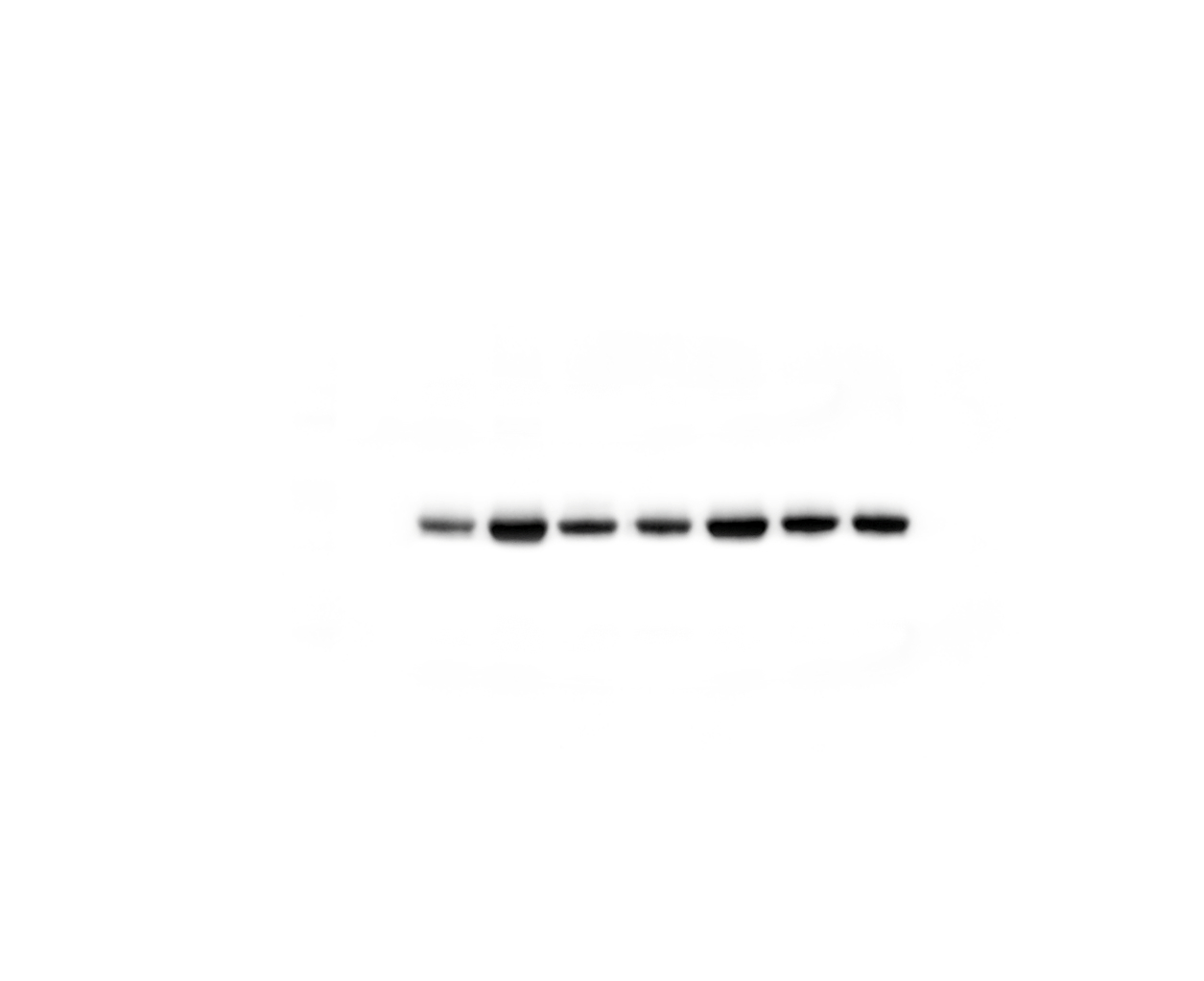

Supplement: Supplementary file 1 — Additional file 1. Protein bands in Western blot. [file 40001_2023_1288_MOESM1_ESM.zip › original image for western blot/Figure 5F/PRKCSH.jpg]

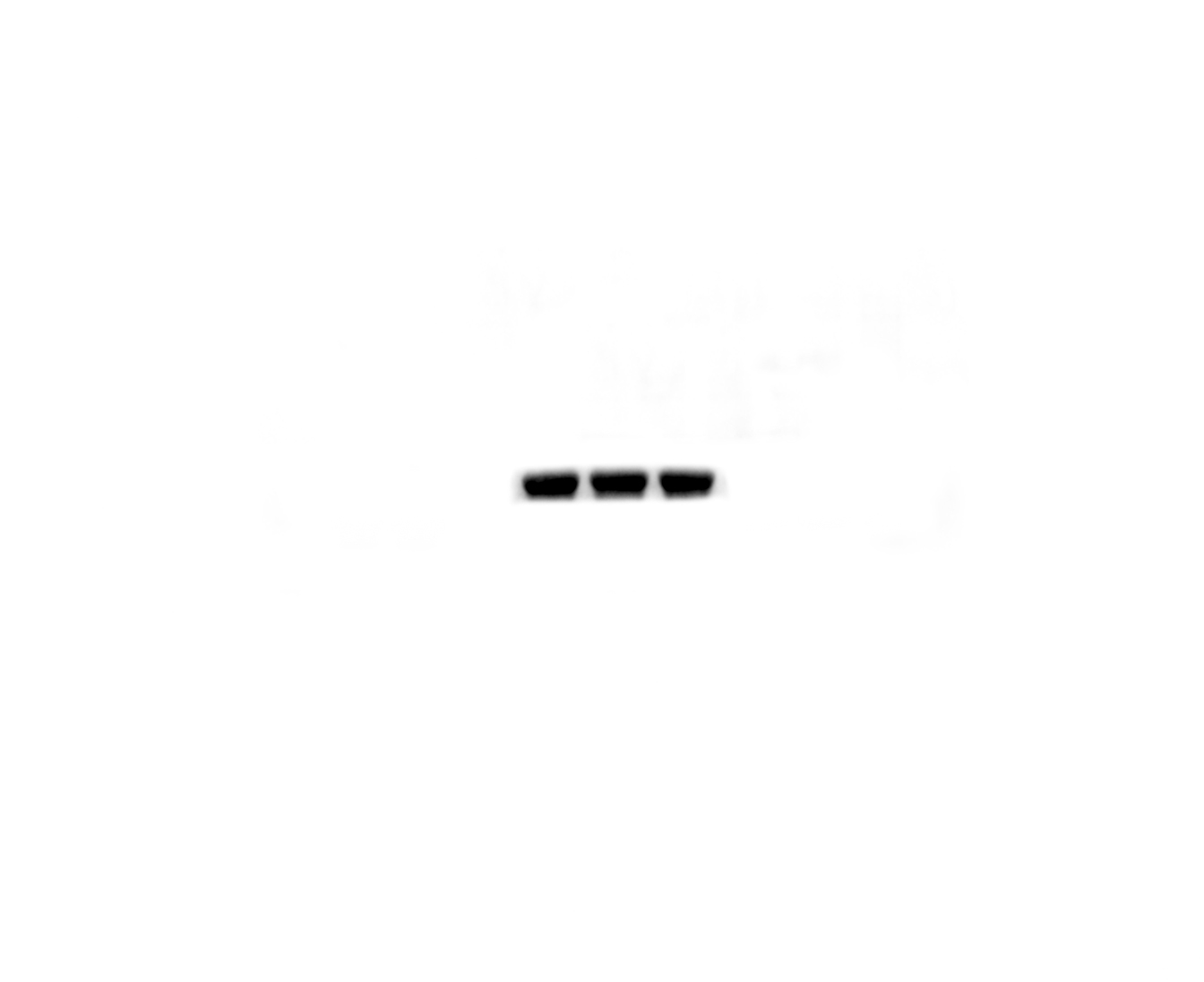

Supplement: Supplementary file 1 — Additional file 1. Protein bands in Western blot. [file 40001_2023_1288_MOESM1_ESM.zip › original image for western blot/Figure 6A/GAPDH.jpg]

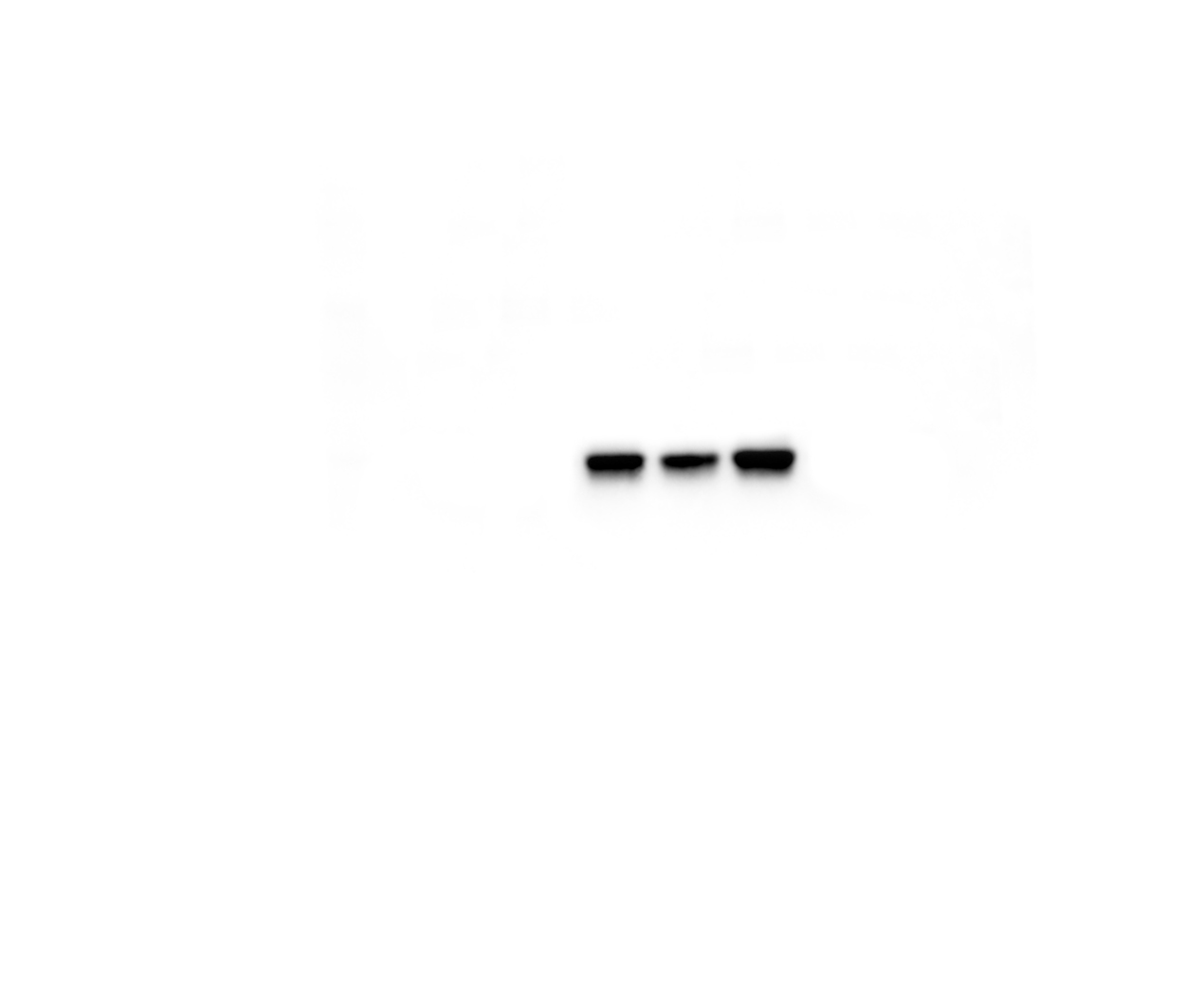

Supplement: Supplementary file 1 — Additional file 1. Protein bands in Western blot. [file 40001_2023_1288_MOESM1_ESM.zip › original image for western blot/Figure 6A/PRKCSH.jpg]

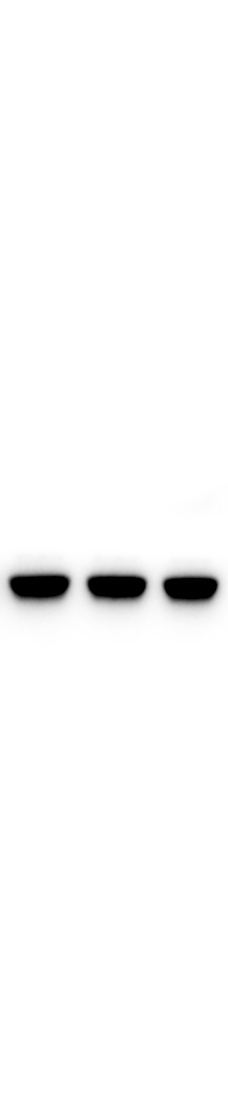

Supplement: Supplementary file 1 — Additional file 1. Protein bands in Western blot. [file 40001_2023_1288_MOESM1_ESM.zip › original image for western blot/Figure 6I/GAPDH.JPG]

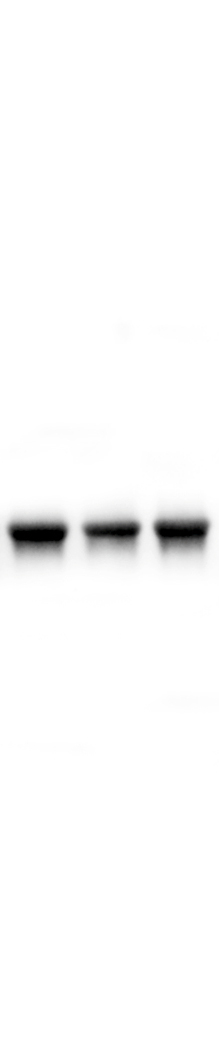

Supplement: Supplementary file 1 — Additional file 1. Protein bands in Western blot. [file 40001_2023_1288_MOESM1_ESM.zip › original image for western blot/Figure 6I/HK2.jpg]

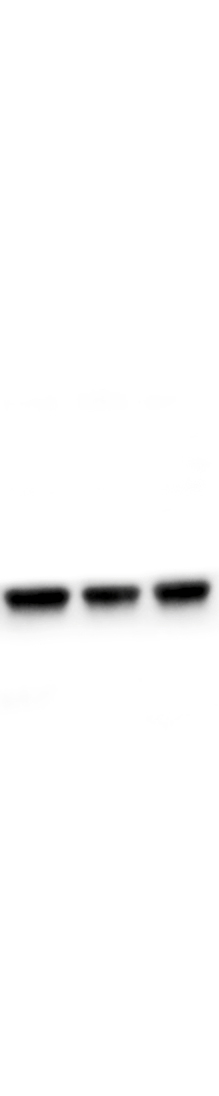

Supplement: Supplementary file 1 — Additional file 1. Protein bands in Western blot. [file 40001_2023_1288_MOESM1_ESM.zip › original image for western blot/Figure 6I/PKM2.jpg]

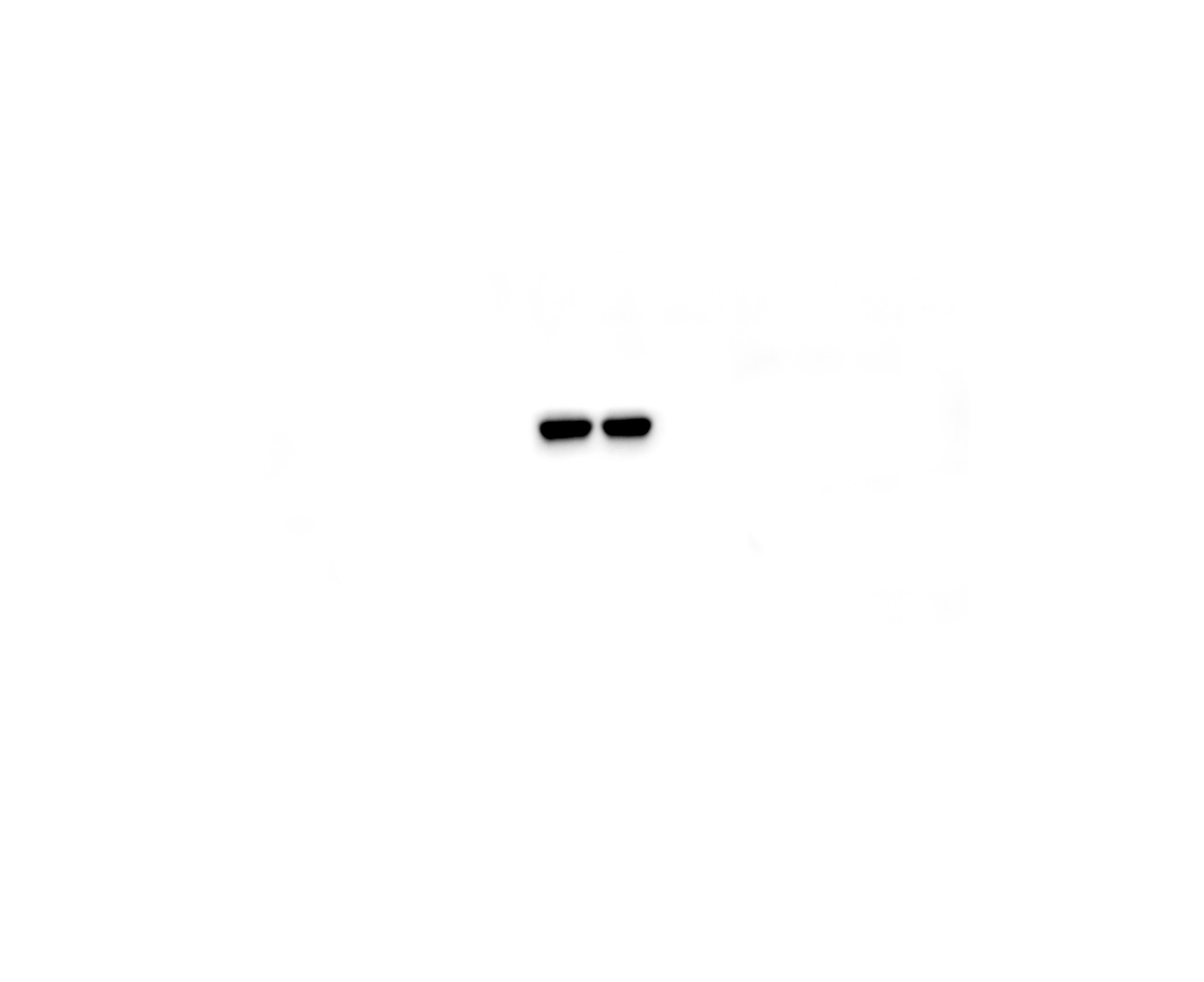

Supplement: Supplementary file 1 — Additional file 1. Protein bands in Western blot. [file 40001_2023_1288_MOESM1_ESM.zip › original image for western blot/Figure 7E/GAPDH.jpg]

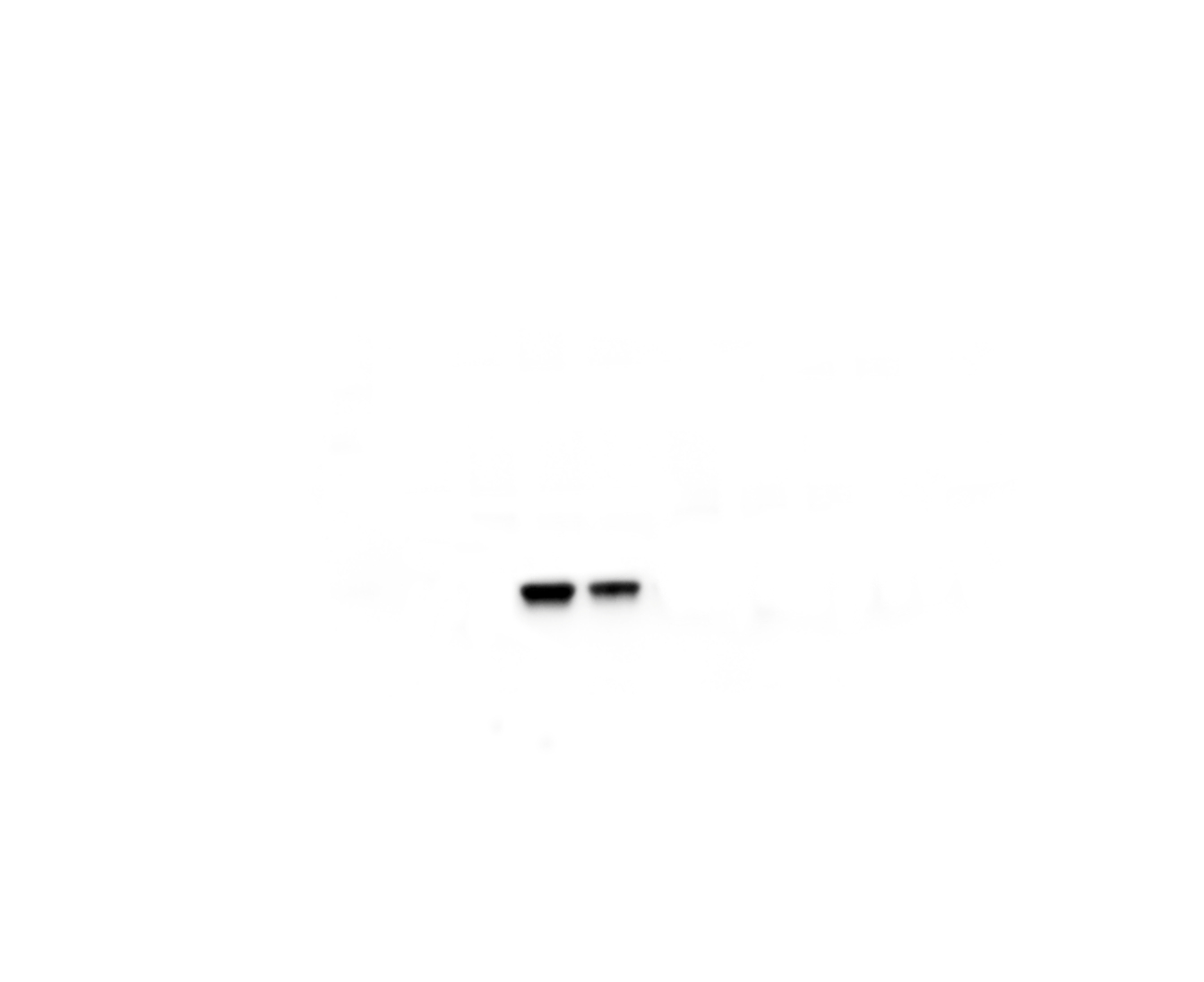

Supplement: Supplementary file 1 — Additional file 1. Protein bands in Western blot. [file 40001_2023_1288_MOESM1_ESM.zip › original image for western blot/Figure 7E/Ki-67.jpg]

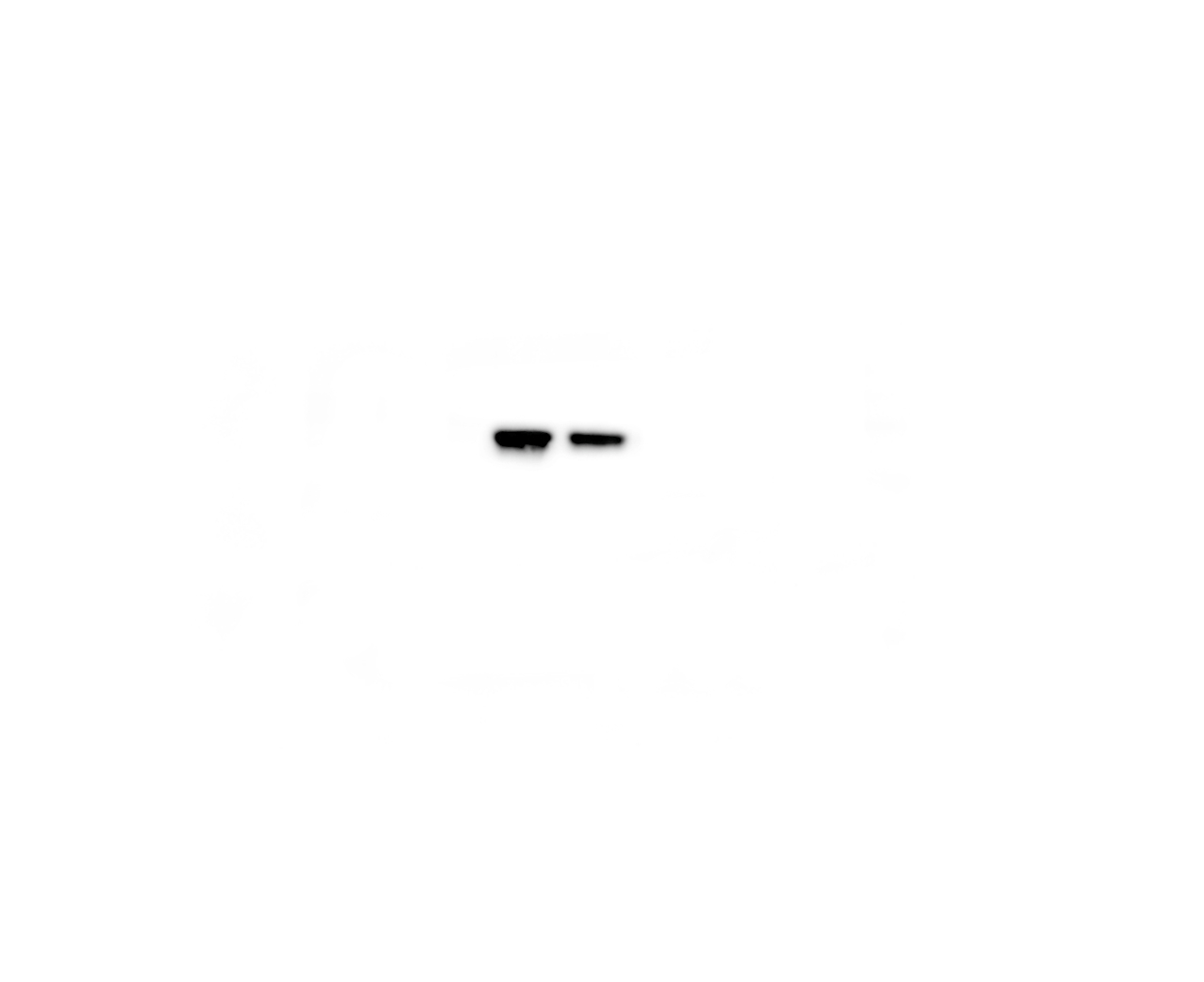

Supplement: Supplementary file 1 — Additional file 1. Protein bands in Western blot. [file 40001_2023_1288_MOESM1_ESM.zip › original image for western blot/Figure 7E/PRKCSH.jpg]

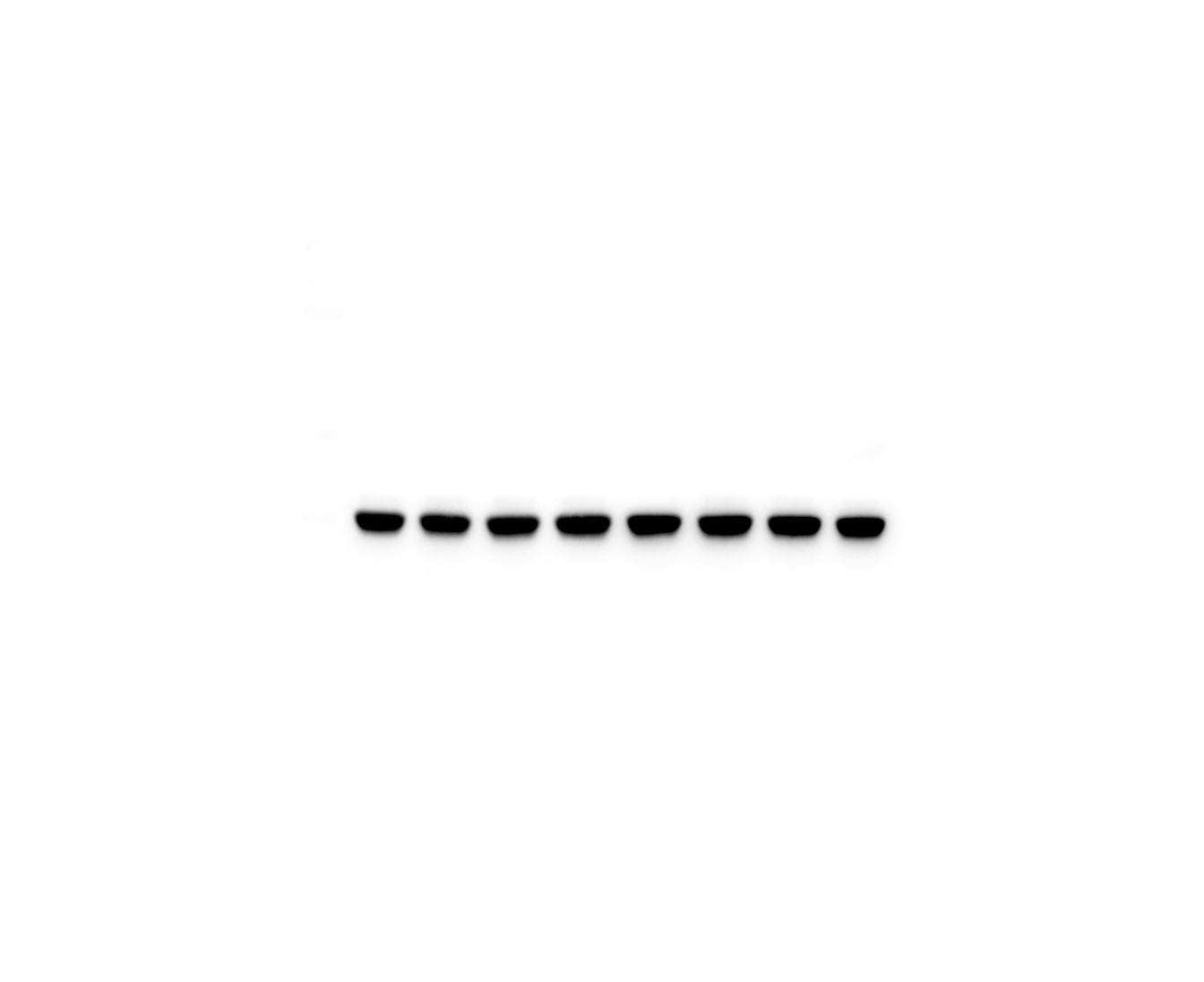

Supplement: Supplementary file 1 — Additional file 1. Protein bands in Western blot. [file 40001_2023_1288_MOESM1_ESM.zip › original image for western blot/Figure2I-Figure4I-Figure6I-GAPDH.jpg]

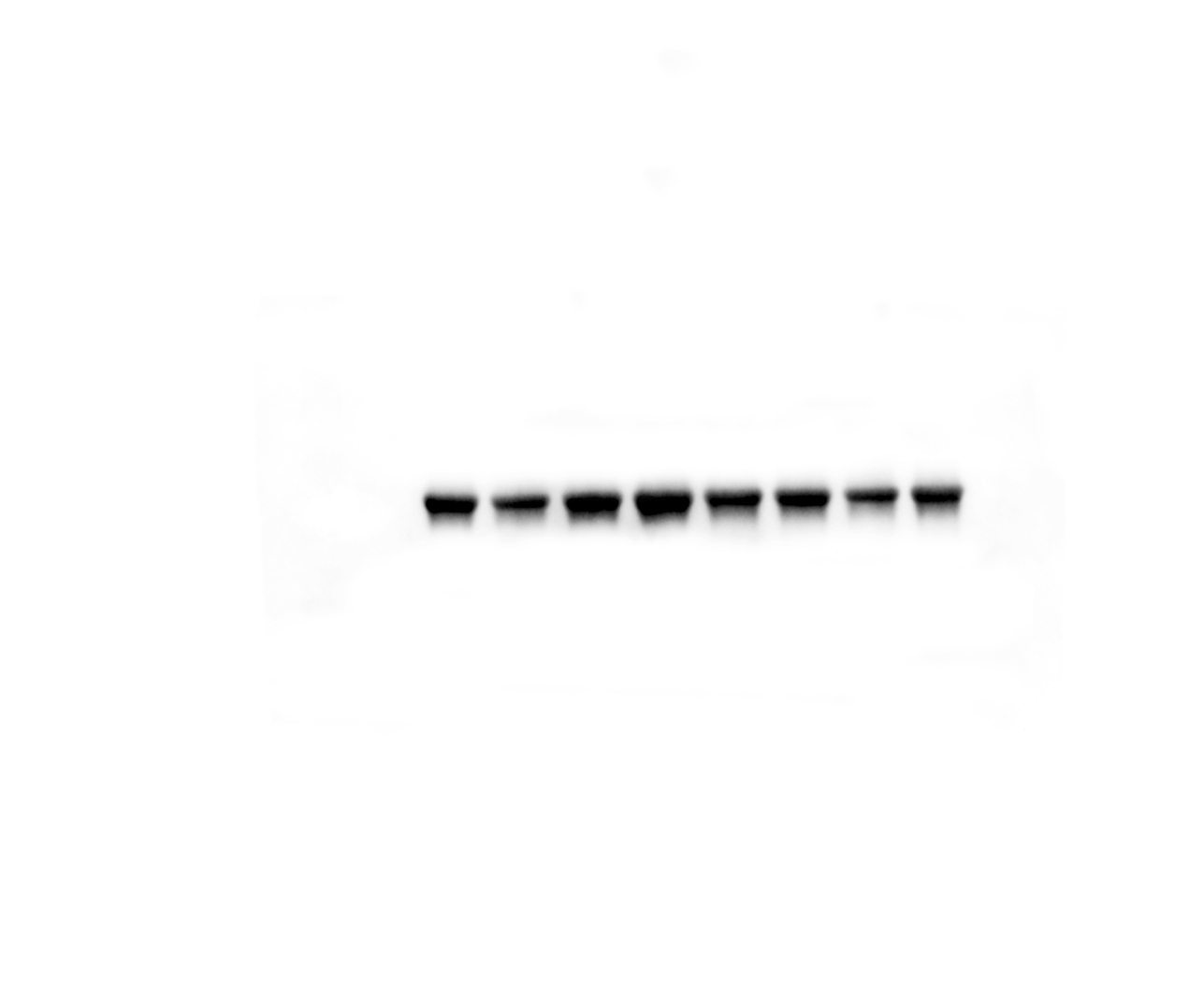

Supplement: Supplementary file 1 — Additional file 1. Protein bands in Western blot. [file 40001_2023_1288_MOESM1_ESM.zip › original image for western blot/Figure2I-Figure4I-Figure6I-HK2.jpg]

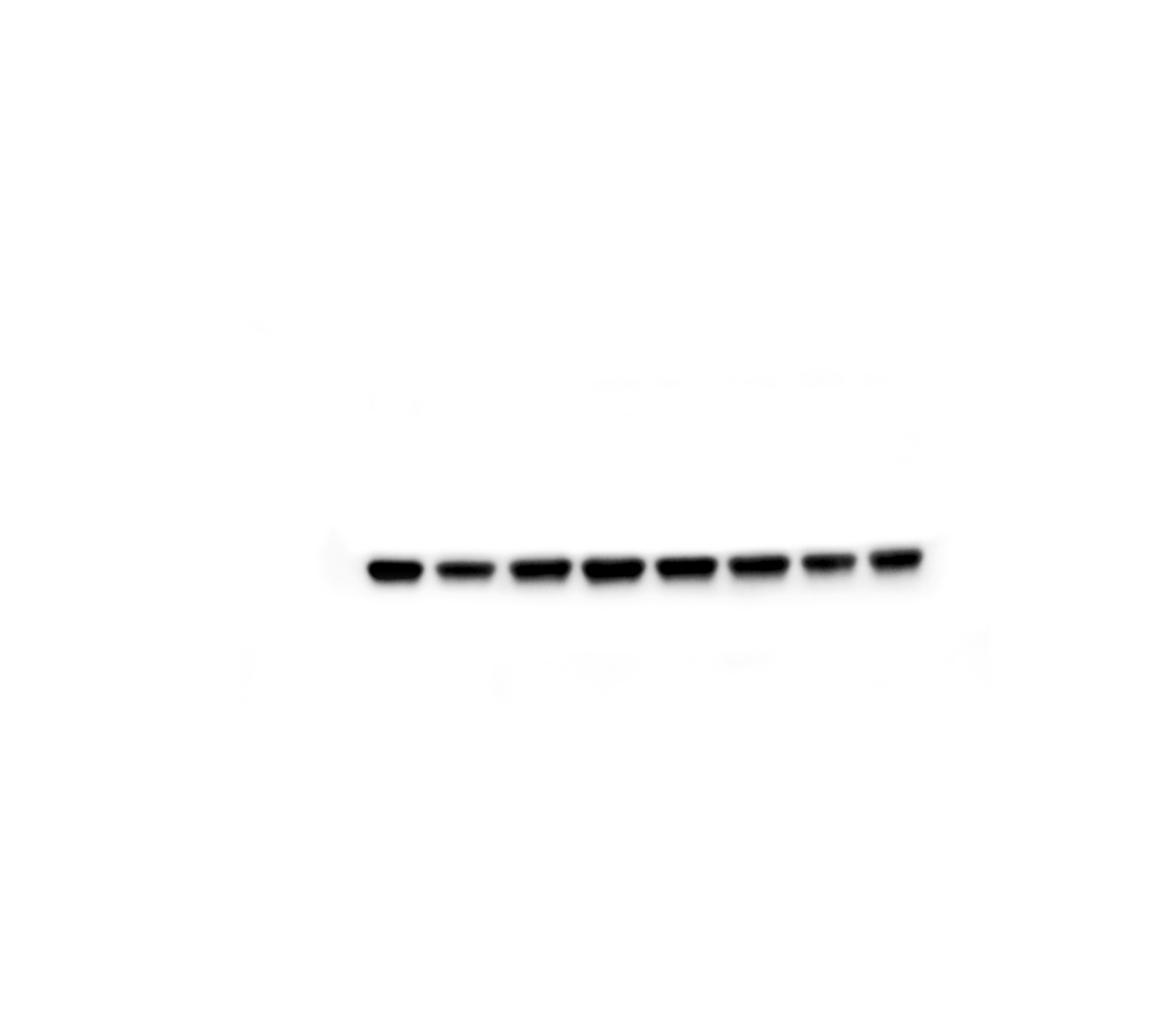

Supplement: Supplementary file 1 — Additional file 1. Protein bands in Western blot. [file 40001_2023_1288_MOESM1_ESM.zip › original image for western blot/Figure2I-Figure4I-Figure6I-PKM2.jpg]
